# Apoptosis Pathways Triggered by a Potent Antiproliferative Hybrid Chalcone on Human Melanoma Cells

**DOI:** 10.3390/ijms222413462

**Published:** 2021-12-15

**Authors:** Irene Rodríguez, Ester Saavedra, Henoc del Rosario, Juan Perdomo, José Quintana, Filippo Prencipe, Paola Oliva, Romeo Romagnoli, Francisco Estévez

**Affiliations:** 1 Departamento de Bioquímica y Biología Molecular, Instituto Universitario de Investigaciones Biomédicas y Sanitarias, Universidad de Las Palmas de Gran Canaria, 35016 Las Palmas de Gran Canaria, Spain; irene.rodriguez118@alu.ulpgc.es (I.R.); ester.saavedra102@alu.ulpgc.es (E.S.); henoc.del101@alu.ulpgc.es (H.d.R.); juan.perdomo@ulpgc.es (J.P.); jose.quintana@ulpgc.es (J.Q.); 2 Instituto Canario de Investigación del Cáncer (ICIC), 35016 Las Palmas de Gran Canaria, Spain; 3 Dipartimento di Scienze Chimiche, Farmaceutiche ed Agrarie, Via L. Borsari 46, 44121 Ferrara, Italy; prnfpp@unife.it (F.P.); lvopla@unife.it (P.O.); rmr@unife.it (R.R.)

**Keywords:** apoptosis, caspases, cytotoxicity, hybrid chalcones, extracellular signal-regulated kinases, melanoma, mitogen-activated protein kinase, reactive oxygen species

## Abstract

The World Health Organization reported that approximately 324,000 new cases of melanoma skin cancer were diagnosed worldwide in 2020. The incidence of melanoma has been increasing over the past decades. Targeting apoptotic pathways is a potential therapeutic strategy in the transition to preclinical models and clinical trials. Some naturally occurring products and synthetic derivatives are apoptosis inducers and may represent a realistic option in the fight against the disease. Thus, chalcones have received considerable attention due to their potential cytotoxicity against cancer cells. We have previously reported a chalcone containing an indole and a pyridine heterocyclic rings and an α-bromoacryloylamido radical which displays potent antiproliferative activity against several tumor cell lines. In this study, we report that this chalcone is a potent apoptotic inducer for human melanoma cell lines SK-MEL-1 and MEL-HO. Cell death was associated with mitochondrial cytochrome *c* release and poly(ADP-ribose) polymerase cleavage and was prevented by a non-specific caspase inhibitor. Using SK-MEL-1 as a model, we found that the mechanism of cell death involves (i) the generation of reactive oxygen species, (ii) activation of the extrinsic and intrinsic apoptotic and mitogen-activated protein kinase pathways, (iii) upregulation of TRAIL, DR4 and DR5, (iv) downregulation of p21^Cip1/WAF1^ and, inhibition of the NF-κB pathway.

## 1. Introduction

Among the different kinds of cancer, melanoma is the deadliest form of skin cancer for its aggressiveness and resistance to current therapies. The number of new cases of melanoma reported by the World Health Organization was approximately 324,000 with an associated 57,000 deaths worldwide in 2020 [1]. It is estimated that 196,060 new cases of melanoma will be diagnosed in the United States in 2021 [2,3]. Melanoma is the third most commonly diagnosed cancer in the group aged 20 to 39 years [4]. Current treatment for melanoma include, in addition to an adequate surgical excision, chemotherapy, radiotherapy, immunotherapy and targeted therapy [5,6,7,8]. However, many of these treatments are expensive, toxic, and less effective in treating the disease due to the appearance of resistance especially in the metastatic form [9]. Moreover, the introduction of new therapies such as immune checkpoint inhibitors and targeted therapies for metastatic melanoma has allowed a decrease in death rates for melanoma of 6.4% per year only between 2013 and 2017 in the United States [10,11]. Despite progress in treatments some patients are refractory to current therapies or develop resistance. Therefore, there is an urgent need to develop and improve potential new therapies in patients with melanoma.

The evasion of apoptosis is considered one of the hallmarks of cancer and there is a great interest and need for the development of new potential anticancer agents for use in clinical oncology that may overcome different types of resistance [12]. Apoptosis is a kind of cell death which is mediated by the activation of a class of cysteine-aspartic proteases, known as caspases [13]. These enzymes are responsible for many of the morphological and biochemical features of regulated cell death, including phosphatidylserine externalization, chromatin condensation, DNA fragmentation, and formation of apoptotic bodies. Two main apoptotic pathways have been described. The intrinsic pathway involves mitochondrial cytochrome *c* release to the cytosol and caspase-9 activation [14]. The extrinsic pathway is mediated by death receptors and involves caspase-8 activation [15]. Both caspase-8 and caspase-9 can catalyze the proteolytic activation of caspase-3 and caspase-7 which are responsible for cell demolition.

One of the most relevant aspects in the regulation of apoptosis and survival is the signaling by the mitogen-activated protein kinases (MAPKs) and the phosphatidylinositide 3-kinase (PI3K)/AKT pathways. The main groups of MAPKs include the extracellular signal-regulated kinases (ERKs) that also include ERK5, the c-Jun NH_2_ terminal kinases (JNKs) and the p38 MAP kinases (p38^MAPK^). Mitogens and growth factors activate the ERK cascade, while the JNK and p38^MAPK^ pathways are involved in apoptosis induction by diverse stimuli. Melanomas frequently exhibit mutations in the RAS/RAF/MEK/ERK pathway. The B-RAF^V600E^ mutation which is caused by the substitution of valine for glutamate at position 600 has been found in 50% cutaneous melanoma. This mutation determines that B-RAF is constitutively activated, which continuously stimulates MEK and ERK, leading to the proliferation and survival of melanoma cells [16]. Previous studies have also shown that the ERK and the PI3K/AKT pathways are interconnected in cutaneous melanoma [17]. For this reason, targeting both pathways may be an option in the treatment of melanoma [18].

Alterations in MAPKs and PI3K/AKT pathways may be triggered by reactive oxygen species (ROS). These chemical species are generated by the partial reduction of molecular oxygen and they are involved in the regulation of cell proliferation and apoptosis [19]. Compounds used in chemotherapy in the treatment of cancer are known to increase ROS generation resulting in cell death [20]. Many anticancer agents are inspired in naturally occurring products. Chalcones (1,3-diphenyl-2-propen-1-ones), a class of compounds characterized by the presence of two aromatic rings linked by a three-carbon α,β-unsaturated carbonyl system, have been shown to possess numerous biological activities, including antiproliferative, antiangiogenic, and anticancer properties [21]. This electrophilic α,β-unsaturated carbonyl moiety allows chalcones to act as Michael acceptors and react with cellular nucleophilic residues which may be responsible for covalent modifications of specific target proteins [22]. Both the double bond of the α,β-unsaturated carbonyl and the planar structure geometry have been reported to be essential for the potential anticancer activity of these compounds [23]. Chalcones constituted by an α,β-unsaturated ketone linking two aromatic heterocyclic rings represented by indole and pyridine moieties have attracted a lot of attention as potential anticancer agents. We have recently described different series of α-bromoacryloylamido indolyl pyridinyl propenones that show cytotoxicity against five human cancer cell lines [24]. These hybrid molecules were prepared to combine the α-bromoacryloyl moiety with two series of indole-inspired chalcone analogs, containing an indole derivative and a 3- or 4-pyridine ring, respectively, linked on either side of 2-propen-1-one system. Several human cancer cell lines, including a B cell precursor (NALM-6), myeloid (U-937, K-562), and lymphoid (MOLT-3) leukemia cells, and the SK-MEL-1 melanoma cells, were highly sensitive to the antiproliferative effect of these compounds. The most cytotoxic chalcone, *(E)-2-bromo-N-(1-methyl-3-(3-oxo-3-(pyridin-4-yl)prop-1-en-1-yl)-1H-indol-5-yl)acrylamide* (HY-CHAL), induced apoptosis along with a decrease in mitochondrial membrane potential in human leukemia cells and showed no cytotoxicity against fresh or proliferating peripheral blood mononuclear cells. However, the potential significance of this specific hybrid chalcone in antimelanoma therapy has not been explored to date. The main aim of this work was to investigate whether HY-CHAL is a potential apoptotic inducer in melanoma cells and to elucidate the signal transduction pathways involved in cell death.

## 2. Results and Discussion

### 2.1. The Synthetic Hybrid Chalcone HY-CHAL Decreases the Viability of Human Melanoma Cells

**Figure 1:**
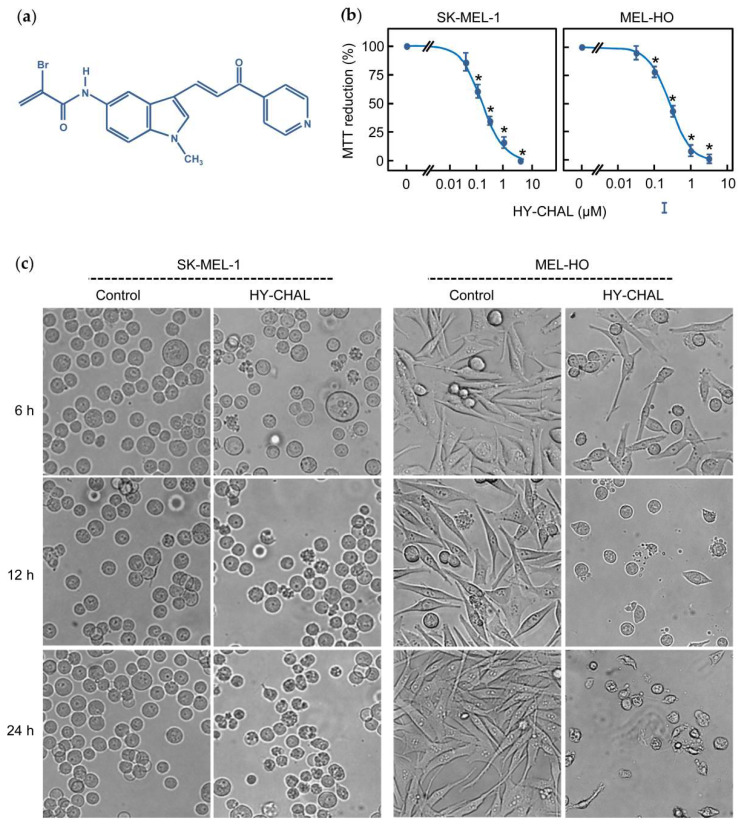
The hybrid chalcone (HY-CHAL) reduced the viability of human SK-MEL-1 and MEL-HO melanoma cells. (**a**) Chemical structure of the synthetic HY-CHAL; (**b**) Dose-response study on MTT reduction (means ± SEs; * indicates significant difference from the control at 0.05 significance level). Cells were incubated in the presence of increasing concentrations of HY-CHAL for 72 h, and thereafter mitochondrial respiratory function was determined by the MTT assay; (**c**) Cells were incubated with 3 μM HY-CHAL for different time periods and images were obtained with an inverted phase-contrast microscope; original magnification 20×.

Recently, we showed that an α-bromoacryloylamido indole-pyridine-chalcone derivative (HY-CHAL, Figure 1a) displayed high antiproliferative activity against human leukemia cells and showed less cytotoxicity (up 0.1 µM) to either fresh or proliferating normal human peripheral blood mononuclear cells. To determine the effect on SK-MEL viability, cells were treated with increasing concentrations of HY-CHAL and evaluated the mitochondrial respiratory function by colorimetric MTT. As expected, HY-CHAL decreased the metabolic activity in a concentration-dependent manner with an IC_50_ value (the concentration that induces a 50% inhibition of cell growth) of 0.15 ± 0.04 µM (Figure 1b). The antiproliferative and cytotoxic effects of HY-CHAL were also explored in an additional melanoma cell line MEL-HO, a human epithelial-like adherent cell line which has been described to contain the BRAFV600E mutation as well as the SK-MEL-1 cells [25]. This mutation leads to permanent mitogen-activated extracellular-signal-regulated kinase kinase (MEK) and extracellular signal-regulated kinase (ERK) activation. As shown in Figure 1b, HY-CHAL decreased the MTT reduction of MEL-HO in a concentration-dependent manner with an IC_50_ value of 0.3 ± 0.1 µM, similar to the value obtained for SK-MEL-1 cells. The effect of HY-CHAL on melanoma cells was further analyzed by phase-contrast microscopy using the most effective concentration (3 µM). As shown in Figure 1c, untreated SK-MEL-1 cells were round and healthy, while cells incubated in the presence of HY-CHAL showed important morphological changes, accompanied by a clear reduction in their number were also observed in the epithelial-like adherent MEL-HO cells. Together, these data indicate cytotoxicity of HY-CHAL against melanoma cells. In the following experiments, concentrations 10–30-fold higher than the antiproliferative IC_50_ values were used in order to identify the primary targets and early mechanism of action of HY-CHAL, since the reported IC_50_ values were determined at 72 h of treatment, while the potential targets were analyzed after a shorter incubation time.

### 2.2. HY-CHAL Induced Cell Death by Apoptosis on Human Melanoma Cells

**Figure 2:**
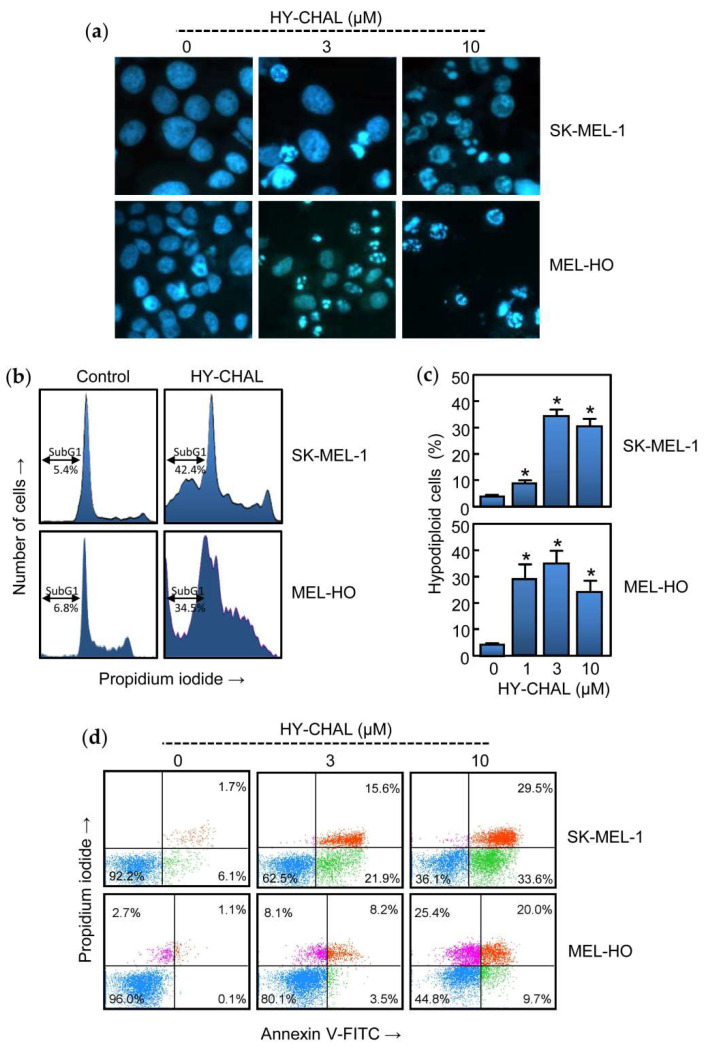
HY-CHAL induced apoptosis in melanoma cells. (**a**) Cells were incubated with increasing concentrations of HY-CHAL for 24 h, stained with bisbenzimide trihydrochloride, and images of representative fields were obtained with a fluorescence microscopy; (**b**) Representative histograms of flow cytometry study. Cells were incubated with 3 µM HY-CHAL, fixed and analyzed by flow cytometry after propidium iodide labeling. Hypodiploid cells (i.e., apoptotic cells) are shown in the region marked with a double edge arrow; (**c**) Cells were treated with increasing concentrations of HY-CHAL and the percentage of hypodiploid cells was determined by flow cytometry. Bars represent the means ± SEs of three independent experiments each were performed in triplicate. * *p* < 0.05, significantly different from untreated control; (**d**) Cells were incubated with the specified concentrations of HY-CHAL for 24 h, stained with annexin V-FITC and propidium iodide and analyzed by flow cytometry. Data shown are representative of three independent experiments with similar results.

To determine whether the cytotoxicity triggered by HY-CHAL was due to regulated cell death induction, treated cells were analyzed by fluorescent microscopy and flow cytometry. As shown in Figure 2a, HY-CHAL induced important morphological changes characteristic of apoptotic cells (condensed and fragmented chromatin) as detected by fluorescent microscopy after Hoechst 33,258 staining. Flow cytometric analyses revealed an increase in the percentage of hypodiploid cells (i.e., apoptotic cells). The histogram shows that the percentage of apoptotic cells increased ~8 fold (5.4% vs. 42.4%) in HY-CHAL-treated SK-MEL-1 cells, after 24 h exposure at a concentration as low as 3 μM. A similar trend was observed in MEL-HO cells. HY-CHAL (3 µM, 24 h) increased ~5-fold the percentage of hypodiploid cells indicating apoptotic cell death (Figure 2b). Dose-response experiments revealed an increase in the percentages of hypodiploid cells dependent on concentration (Figure 2c). In addition, HY-CHAL also led to the exposure of phosphatidylserine on the outside of the plasma membrane, which is an early marker of apoptosis, as detected by Annexin V-FITC staining (Figure 2d). These results demonstrated that HY-CHAL induced apoptosis in SK-MEL-1 and in MEL-HO and this effect was concentration-dependent.

### 2.3. HY-CHAL Induces Cell Death by a Caspase-Dependent Pathway in Human Melanoma Cells

**Figure 3:**
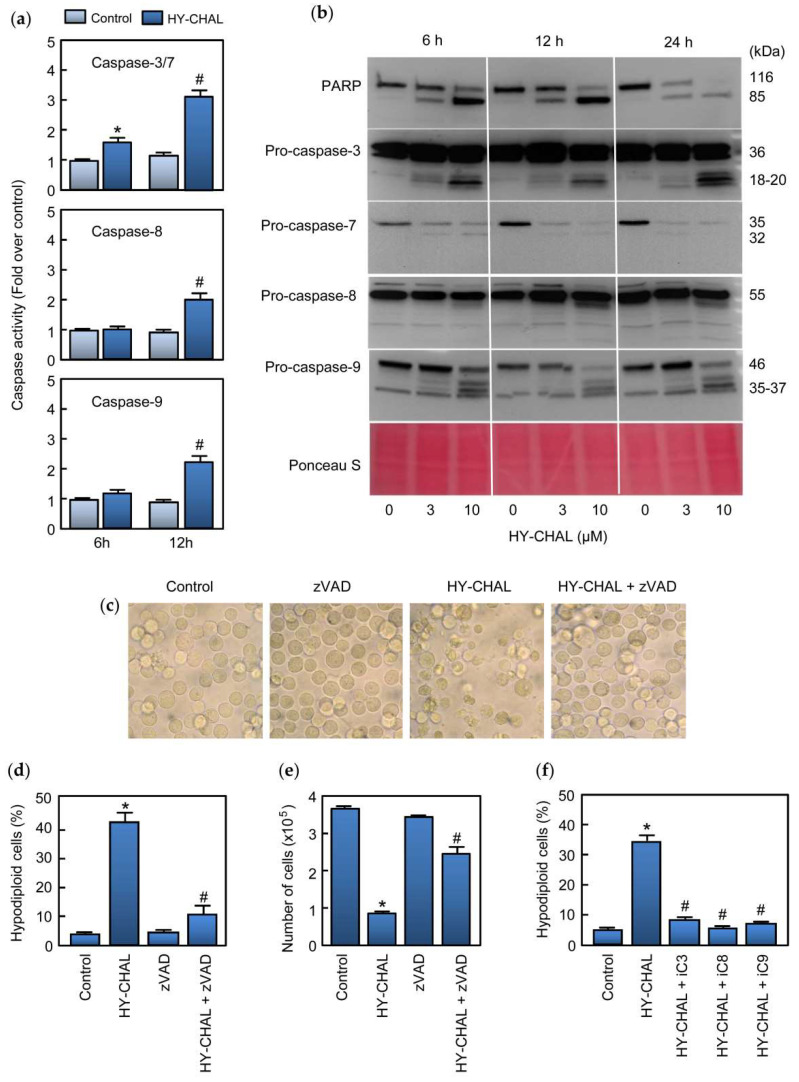
HY-CHAL induced caspase cascade in SK-MEL-1 melanoma cells. (**a**) Cells were treated with 3 μM HY-CHAL for the indicated times and caspase activation was determined using specific colorimetric substrates; (**b**) Cells were treated with the specified concentrations of HY-CHAL and poly(ADP-ribose) polymerase (PARP) cleavage and caspase processing was determined by immunoblotting. The membrane was stained with Ponceau S before antibody detection to control equal protein loading; (**c**) Cells were pretreated with 100 µM z-VAD-fmk for 1 h and then incubated in the absence or in the presence of 3 µM HY-CHAL for 24 h and images were obtained with an inverted phase-contrast microscope; (**d**) Cells were treated as in (**c**) and hypodiploid cells (i.e., apoptotic cells) were analyzed by flow cytometry. Bars represent the mean ± SE of three independent experiments each performed in triplicate. * Indicates *p* < 0.05 for comparison with untreated control. ^#^ Indicates *p* < 0.05 for comparison with HY-CHAL treatment alone; (**e**) Cells were treated as in (**c**) and cell viability was determined by the trypan blue exclusion method using a TC counter; (**f**) Cells were pretreated with the selective caspase inhibitors z-DEVD-fmk (iC3, 50 µM), z-IETD-fmk (iC8, 50 µM) and z-LEHD-fmk (iC9, 50 µM) for 1 h before the addition of 3 µM HY-CHAL and apoptotic cells were quantified as in (**d**).

Caspases are essential for the execution of apoptosis. To determine whether caspase activation was associated with the cell death induced by HY-CHAL, enzymatic analysis of these cysteine proteases was performed in time-course experiments using specific colorimetric tetrapeptide substrates (DEVD-*p*NA, IETD-*p*NA and LEHD-*p*NA for caspase-3/7, caspase-8 and caspase-9, respectively). As shown in Figure 3a, treatment of SK-MEL-1 cells with 3 µM HY-CHAL induced activation of the executioner caspase-3/7 which was detected after 6 h and activity was more pronounced at 12 h of treatment. At this time, an increase of the activity of the initiator caspases, caspase-8 and -9, was also observed.

Processing of caspases was also determined by western blot using specific antibodies against caspases and the DNA repair enzyme poly(ADP-ribose) polymerase (PARP) which is a known caspase-3 and caspase-7 substrate [26]. To this end, SK-MEL-1 cells were incubated with increasing concentrations of HY-CHAL for different time periods and lysates were analyzed by immunoblotting. The results showed PARP cleavage leading to the formation of the characteristic ~85 kDa fragment which was evident after 6 h of treatment with 3 µM HY-CHAL. Using MTT assay, SK-MEL-1 cells viability was 40% at 3 µM for 24 h. Processing of caspases-3 and -7 was also detected by an increase in the corresponding fragments and in the case of caspase-7 by a decrease in the zymogen as well. These results are in accordance with PARP cleavage. Processing of initiator caspases, caspases-8 and -9, which were detected by the generation of the fragments as well as by an important decrease in the corresponding proenzyme in the case of caspase-9 (Figure 3b). In these experiments, Ponceau S staining before antibody detection was used as an alternative loading control. To define which caspases are involved in MEL-HO cell death, the enzymatic activity of cell lysates was analyzed after 24 h of treatment. As shown in Figure S1a (Supplementary materials), dose-response experiments revealed that a low concentration (3 µM) of the chalcone activated the executioner caspase-3/7 activity, as well as the initiator caspases-8 and 9. The processing of caspases was analyzed by immunoblotting. As shown in Figure S1b (Supplementary materials), HY-CHAL stimulated the cleavage of caspase-3 and the initiator caspases (caspase-8 and -9). In accordance with the activation of caspase-3/7 and caspase-3 processing, PARP was effectively cleaved to the 85 kDa fragment.

We evaluated whether the pan-caspase inhibitor z-VAD-fmk could rescue SK-MEL-1 melanoma cells from HY-CHAL-induced cell death. To this end, cells were pretreated with z-VAD-fmk (100 µM) and cultured in the absence or the presence of HY-CHAL for 24 h and visualized with an inverted phase-contrast microscope. As shown in Figure 3c, the cells appeared healthy in the combination group (z-VAD-fmk plus HY-CHAL). In addition, the general inhibitor of caspases was able to block in great part the increase in the percentage of apoptotic cells as determined by flow cytometry and to rescue the cells from death as determined by the trypan blue exclusion method (Figure 3d,e). z-VAD-fmk also blocked HY-CHAL-induced cell death in MEL-HO cells, as visualized by phase-contrast microscopy and determined by flow cytometric analysis of annexin V-FITC and propidium iodide stained cells (Supplementary materials, Figure S1c,d). The results showing that z-VAD-fmk inhibited cell death induced by HY-CHAL indicate that caspase activation has a key role in cell death. To deep knowledge about which specific caspase is involved in HY-CHAL-induced cell death, SK-MEL-1 cells were pretreated with permeable and selective caspase inhibitors for caspase-3/7, -8, and -9. As shown in Figure 3f, not only the caspase-3/7 inhibitor was able to block the increase in the percentage of hypodiploid cells, but also the specific caspase-8 and -9 inhibitors blocked completely cell death triggered by HY-CHAL.

### 2.4. HY-CHAL Down-Regulated the Pro-Survival Bcl-2 Members and Up-Regulated the Pro-Apoptotic Bcl-2 Proteins and Death Receptors and Its Ligand in Human Melanoma SK-MEL-1 Cells

**Figure 4:**
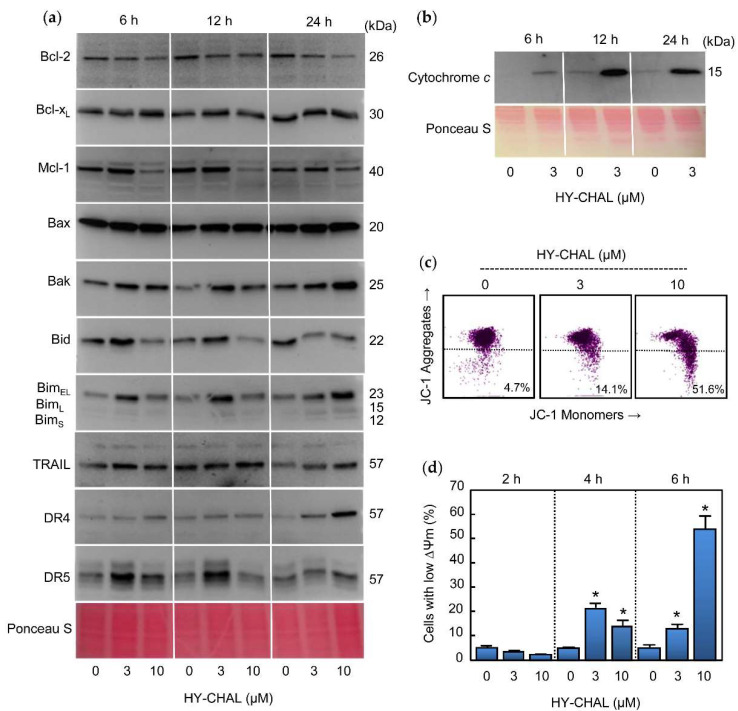
Role of the mitochondria, TRAIL and Death Receptors in the apoptotic cell death triggered by HY-CHAL in SK-MEL-1 melanoma cells. (**a**) Cells were treated with the specified concentrations of HY-CHAL for the indicated times and whole cell lysates were probed with antibodies raised against the indicated Bcl-2 family proteins, TRAIL, and death receptors by immunoblotting. Equal protein loading was controlled by staining the membranes with Ponceau S before the incubation with antibodies (a representative section of the stained membrane is shown); (**b**) Cells were treated with 3 µM HY-CHAL for the indicated times and cytosolic fractions prepared, separated by SDS-PAGE and cytochrome *c* was detected by immunoblotting. Equal protein loading was controlled by staining the membranes with Ponceau S; (**c**) Cells were incubated with the indicated concentrations of HY-CHAL for 6 h and ΔΨ_m_ analyzed by flow cytometry after staining with the JC-1 probe as described in the Experimental Section; (**d**) Cells were incubated in control conditions or in the presence of the indicated concentrations of HY-CHAL for the specified times, and the percentage of cells with reduced ΔΨ_m_ was quantified by flow cytometry using the fluorescent probe JC-1. * Indicates *p* < 0.05 for comparison with untreated control.

The effects of HY-CHAL on B-cell lymphoma-2 (Bcl-2) family proteins expression were evaluated since these proteins control the intrinsic apoptotic pathway and play a crucial role in protecting against cancer. To this end, cells were treated with increasing concentrations of HY-CHAL and different time periods and whole cell lysates were analyzed by immunoblotting (Figure 4a). Among the Bcl-2 proteins with anti-apoptotic activities, the expression of Bcl-2 itself, Bcl-x_L_ (B-cell lymphoma-extra-large), and Mcl-1 (Myeloid cell leukemia 1), one of the most potent anti-apoptotic protein of the Bcl-2 family, were analyzed. There was a decrease in Bcl-2 levels which was evident with 3 µM and 10 µM HY-CHAL after 24 h of treatment. Although 3 µM HY-CHAL slightly upregulated Mcl-1, the levels of this pro-survival factor decreased after 6 h and 12 h of treatment with 10 µM HY-CHAL. In contrast, there were no changes in Bcl-x_L_ levels at any time assayed.

Expression of the pro-apoptotic Bcl-2 proteins Bax (Bcl-2-associated X protein) and Bak (Bcl-2 antagonist killer) were analyzed since they are involved in the increase in permeability of the outer mitochondrial membrane and the release of proteins from the intermembrane space into the cytoplasm [27], which lead the subsequent initiation of the caspase cascade. As shown in Figure 4a, an increased levels of Bax and Bak were observed for all times assayed.

The BH3-only proteins of the Bcl-2 family are involved in the inhibition of pro-survival Bcl-2 proteins as well as in the direct activation of Bax and Bak. Therefore, the mechanism of action HY-CHAL was explored in relation to changes in the levels of the BH3-only proteins Bid (BH3-interacting domain death agonist) and Bim (Bcl-2 interacting mediator of cell death). Time-course and dose-response experiments revealed up-regulation of BimEL isoform at 6 h of treatment with 3 µM HY-CHAL and all three major Bim isoforms (BimEL, BimL, and BimS) increased at 24 h of treatment with 3 and 10 µM HY-CHAL. Among the three Bim isoforms generated by alternative splicing, the shortest form, BimS, is the most cytotoxic and is generally only transiently expressed during apoptosis [28]. Slight increases in Bid levels were observed at 6 h and 12 h with 3 µM HY-CHAL, while there was a decrease at the same times with the highest concentration of the compound and at 24 h with both assayed concentrations, in accordance with the caspase-8 activation. Although we were unable to detect truncated Bid, the decrease in full-length Bid levels is probably a consequence of Bid cleavage by caspase-8.

Since HY-CHAL induced caspase-8 activation, this could be caused by TRAIL (tumor necrosis factor-related apoptosis-inducing ligand) and/or Death Receptors as DR4 (Death Receptor 4) and DR5 (Death Receptor 5) up-regulation. To test this hypothesis, cells were treated with increasing concentrations of compound for the specified times and TRAIL, DR4, and DR5 were analyzed by Western blot. As shown in Figure 4a, a low concentration of HY-CHAL (3 µM) induced up-regulation of DR5 at 6 h and 12 h, whereas DR4 and TRAIL only slightly increased and this was more evident after 24 h of treatment. These results indicate that Death Receptors were differentially up-regulated following different kinetics.

### 2.5. HY-CHAL Induced Cytochrome c Release and Mitochondrial Membrane Potential Depolarization in Human Melanoma Cells

The release of cytochrome *c* of the mitochondrial intermembrane space to cytoplasm is a key event in the intrinsic pathway of cell death. To examine whether HY-CHAL-induced apoptosis involves the release of cytochrome *c* from intermembrane space to cytoplasm, time-course experiments were performed and cytosolic fractions were analyzed by immunoblotting. As shown in Figure 4b, a significant increase in the quantity of cytochrome *c* in the cytosol was detected at all times assayed after treatment with 3 µM HY-CHAL. The mechanism of cell death in MEL-HO was also examined to investigate whether it is involved in the mitochondrial cytochrome *c* release. The results revealed a significant increase in the amount of this hemoprotein in the cytosol, even after treatment with a concentration as low as 1 µM of compound (Supplementary materials, Figure S1b). Since the mitochondrial membrane potential (ΔΨm) dissipation is also a crucial event in the mitochondrial pathway, it was also interesting to investigate whether a disruption of the ΔΨm was associated with the release of cytochrome *c*. To this end, cells were incubated for 6 h with increasing concentrations of HY-CHAL and analyzed by flow cytometry using the fluorescent probe JC-1. As shown in Figure 4c, the chalcone induced a significant loss of ΔΨm, which suggests that the dissipation of the mitochondrial membrane potential might be involved in the mechanism of cell death. Maximal levels of reduced ΔΨm were observed after treatment with 10 µM of compound for 6 h (Figure 4d). A similar trend was observed in MEL-HO cells. HY-CHAL induced a fast dissipation of ΔΨm and maximal levels of reduced ΔΨm were detected with the highest concentration of chalcone (Supplementary materials, Figure S1e,f). In summary, these results showed that HY-CHAL induced apoptosis in SK-MEL-1 and MEL-HO cells which were associated with the release of cytochrome *c* and activation of the extrinsic and intrinsic apoptotic pathway of cell death.

### 2.6. HY-CHAL Activated the Mitogen-Activated Protein Kinase Pathway and Inhibited AKT in Human Melanoma SK-MEL-1 Cells

**Figure 5:**
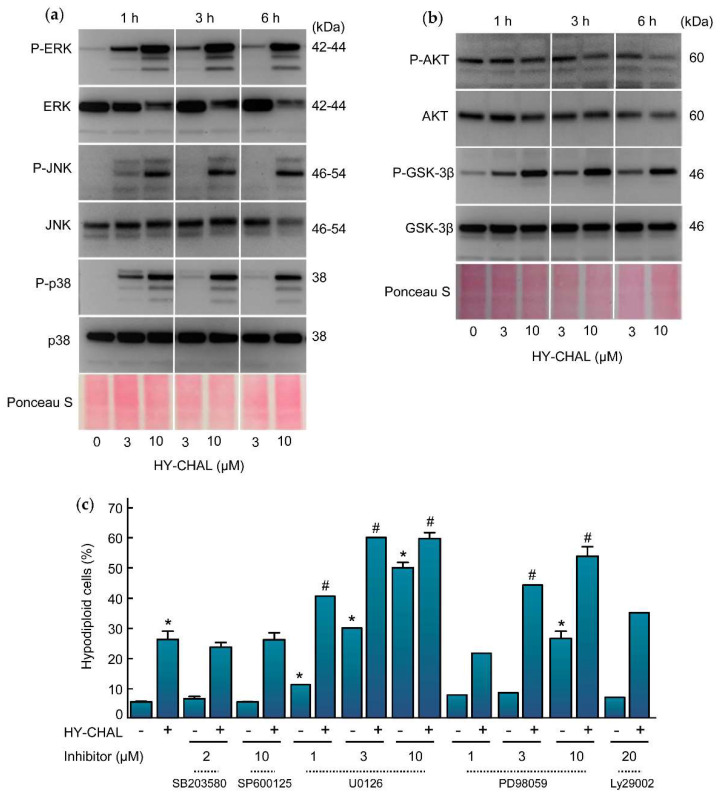
Effect of HY-CHAL on MAPK and AKT pathways. (**a**) Time-dependent phosphorylation of ERK1/2, JNK/SAPK and p38^MAPK^ and (**b**) of AKT and GSK-3β by HY-CHAL. Cells were incubated with the concentrations specified for the time periods shown. Lysates were analyzed on Western blots probed with specific antibodies to ascertain the phosphorylation of MAPKs, AKT, and GSK-3β. Membranes were stripped and reprobed with specified antibodies as loading controls. Equal protein loading was also controlled by staining the membrane with Ponceau S; (**c**) Effect of MAPKs and PI3K inhibitors on HY-CHAL-induced cell death. Cells were preincubated with the p38^MAPK^ inhibitor SB203580 (2 µM), the JNK/SAPK inhibitor SP600125 (10 µM), the indicated concentrations of the MEK1/2 inhibitors U0126 and PD98059 or the PI3K inhibitor LY294002 (20 µM) and then treated with 3 µM HY-CHAL for 24 h. The percentages of hypodiploid cells were determined by flow cytometry after propidium iodide staining. Bars represent means ± SEs of two independent experiments performed in triplicate. * Indicates *p* < 0.05 for comparison with untreated control. ^#^ Indicates *p* < 0.05 for comparison with HY-CHAL treatment alone.

The effect of HY-CHAL on mitogen-activated protein kinases (MAPK) pathways was also evaluated due to their implication in cell proliferation, survival, and death. Specifically, the activation of the MAPK pathway is a central step in melanoma pathogenesis [29]. To this end, SK-MEL-1 cells were treated with increasing concentrations of chalcone for different time periods and activation of MAPKs was determined by western blot using specific antibodies. As shown in Figure 5a, HY-CHAL induced the phosphorylation of ERK1/2, JNK/SAPK and p38^MAPK^. All MAPKs were fast activated starting at 1 h and remained elevated for up to at least 6 h, following similar kinetics and dependent on concentration. Interestingly, there was a fast downregulation of total ERK1/2 after treatment with 10 µM HY-CHAL. In addition to the MAPK pathway, the phosphoinositide 3-kinase (PI 3-kinase) pathway can also be activated by interactions between receptor tyrosine kinases and their ligands. Protein kinase B (PKB/AKT) is a downstream target of growth factor receptor activation and promotes cell survival. To investigate the role of AKT in the mechanism of cell death, the effect of HY-CHAL on phosphorylation status on Ser473, which is involved in its activation, was analyzed. As shown in Figure 5b, phospho-AKT levels were high on SK-MEL-1 cells and HY-CHAL reduced AKT phosphorylation in a time-dependent manner as detected by immunoblotting.

AKT is involved in cell cycle regulation by preventing glycogen synthase kinase-3β (GSK-3β)-mediated phosphorylation and degradation of cyclin D1 and by negatively regulating the cyclin-dependent kinase inhibitors p27 and p21 [30,31]. GSK-3β is a protein serine/threonine kinase and a critical downstream component of the PI3 kinase/AKT cell survival pathway whose activity can be inhibited by AKT-mediated phosphorylation at Ser9 [32]. To explore the effect of HY-CHAL on the activity of GSK-3β we performed time-course and dose-response experiments and determined the levels of phosphoSer9 GSK-3β which is indicative of inactivation. As shown in Figure 5b, there was a fast (1 h) phosphorylation of GSK-3β even at concentrations as low as 3 µM HY-CHAL.

To investigate the potential impact of the MAPK and PI-3K/AKT cascades in the signal transduction pathway of cell death we used specific inhibitors (Figure 5c). The results indicated that activation of both, JNK/SAPK and p38^MAPK^, is not involved in cell death since the specific inhibitors SP600125 (10 µM) and SB203580 (2 µM) were unable to block the increase in the percentage of hypodiploid cells triggered by HY-CHAL. In contrast, the MEK inhibitors PD98059 and U0126 amplified cell death. These results indicate that cell death triggered by HY-CHAL is enhanced by MEK inhibitors and is independent on JNK/SAPK and p38^MAPK^. Inhibition of phosphoinositide 3-kinase pathway with LY294002 did not affect significantly the increase in the percentage of hypodiploid cells, revealing that the PI3K/AKT pathway is not involved in the induction of apoptosis.

### 2.7. HY-CHAL Downregulated β-Catenin, c-Myc and p21^Cip1/WAF1^ and Inhibited NF-κB Pathway in Human Melanoma SK-MEL-1 Cells

**Figure 6:**
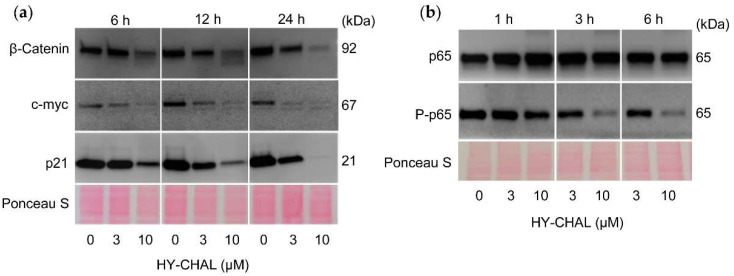
HY-CHAL downregulated β-catenin, c-myc and p21^Cip1/WAF1^, and inhibited NF-κB. (**a**,**b**) Cells were treated with the specified concentrations of HY-CHAL for the indicated times and cell lysates were probed with the specified antibodies. Equal loading was controlled by staining the membrane with Ponceau S.

The effect of HY-CHAL on the β-catenin levels was investigated, since the Wnt/β-catenin pathway plays a key role in the regulation of proliferation and apoptosis in melanoma cells [33,34]. SK-MEL-1 cells express nuclear β-catenin under resting conditions and treatment with HY-CHAL reduced the levels of this transcription factor in a time- and concentration-dependent manner (Figure 6a). This result is surprising in terms of the effects of HY-CHAL on GSK-3β enzyme activity (see Figure 5b). GSK-3β is part of the multiprotein destruction complex which regulates the turnover of the free pool of cytoplasmic β-catenin. Targeting β-catenin by phosphorylation determines its proteasomal degradation. Therefore, it would be expected that the GSK-3β inhibition would block β-catenin degradation and allow its migration to the nucleus to activate the transcription of specific pro-oncogenic proteins. A possible explanation could be the existence of different pools of this protein kinase that would reflect an imbalance between oncogenic (nuclear) and pro-apoptotic (cytoplasmic) GSK-3β, as previously reported [35]. The results showed a clear downregulation of β-catenin starting at 6 h of treatment with 10 µM HY-CHAL. This effect may be caused by proteolytic cleavage of β-catenin and could be related to caspase-3 activation. Additional studies are necessary to confirm the cleavage of β-catenin by this executioner caspase as proposed previously [36,37,38,39,40,41].

Next, we examined the level of the transcription factor c-myc, an end product of the Wnt signaling pathway that is regulated by β-catenin/Tcf-Lef transactivation. To this end, cells were treated during different time periods and with increasing concentrations of HY-CHAL. As shown in Figure 6a, c-myc levels were downregulated and this effect was time- and concentration-dependent. The search for compounds able to suppress c-myc expression is of great interest since this transcription factor regulates the expression of many genes involved in multiple cellular processes including cell cycle [42], plays a key role in the development of a wide variety of cancers and it is upregulated in approximately 70% of human tumors [43,44]. Previous work has also described that the downregulation of c-myc increases the sensitivity of melanoma cells to cisplatin [45].

The cyclin-dependent kinase inhibitor p21^Cip1/WAF1^ may cause cell cycle arrest but also it may act as an inhibitor of apoptosis. For example, the reduction of endogenous p21^Cip1/WAF1^ expression in cells that express high levels, such as MCF-7 cells, attenuates the growth arrest and promotes cell death [46]. In addition, it seems that the normal function of p21^Cip1/WAF1^ is to protect thymic tumor cells against apoptosis, since loss of p21^Cip1/WAF1^ enhances the apoptotic response [47]. As shown in Figure 6a, SK-MEL-1 cells express high levels of p21^Cip1/WAF1^ and the treatment with HY-CHAL decreased the levels in a time- and concentration-dependent manner. p21^Cip1/WAF1^ may be a target of caspase-3 which is activated after treatment with HY-CHAL, as shown above. Previous studies have demonstrated that human cancer cell lines treated with DNA-damaging agents undergo cell cycle arrest mediated by p21^Cip1/WAF1^ and this was followed by apoptosis after caspase-3-mediated cleavage of this cell cycle inhibitor [48]. These authors suggested that the inactivation of p21^Cip1/WAF1^ mediated by caspase-3 is a consequence of the removal of the C-terminal domain, which is essential for its interaction with the proliferating cells nuclear antigen and the nuclear localization. In addition, it has been proposed that compounds that downregulate p21^Cip1/WAF1^ may improve the action of anticancer agents, since the loss of p21^Cip1/WAF1^ usually enhances the sensitivity of tumor cells to apoptosis induced by different chemotherapeutic drugs [49].

The transcription factor NF-κB is a potential target in antimelanoma therapy because it is constitutively activated in this cancer [50] and regulates the expression of genes involved in cell proliferation, survival, angiogenesis, metastasis, and inhibition of apoptosis [51]. To investigate the effect on NF-κB, cells were treated for different time periods with increasing concentrations of HY-CHAL and total p65 and phospho-p65 were determined by western blot. As shown in Figure 6b, SK-MEL-1 cells showed basal phosphorylation of p65 indicating a basal activity of this transcription factor. Phospho-p65 levels were decreased by HY-CHAL, while total p65 amounts remained unchanged, indicating inhibition of the NF-κB canonical pathway.

### 2.8. HY-CHAL Increased Reactive Oxygen Species (ROS) Generation and Glutathione Blocked ROS Formation and Cell Death in Human SK-MEL-1 Melanoma Cells

**Figure 7:**
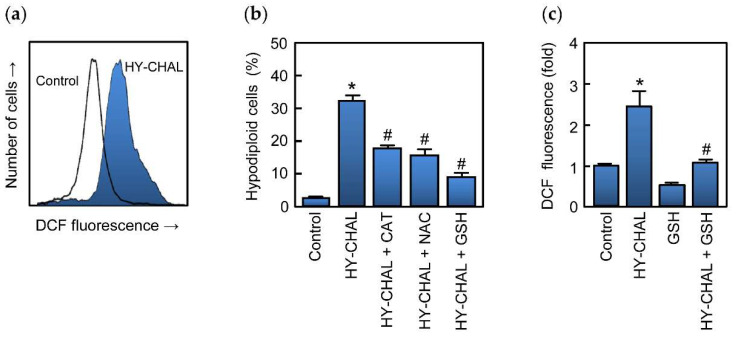
Role of ROS in HY-CHAL-induced cell death. (**a**) Representative histogram obtained by flow cytometry of the fluorescence of oxidized H_2_DCF after treatment of SK-MEL-1 with 3 μM HY-CHAL for 1 h; (**b**) Cells were preincubated with catalase (500 units/mL), *N*-acetyl-L-cysteine (5 mM), or glutathione (5 mM) and then treated with HY-CHAL (3 µM) for 24 h and thereafter the percentage of hypodiploid cells was determined by flow cytometry; (**c**) Fluorescence obtained by the oxidation of H_2_DCF of cells preincubated with glutathione (5 mM) for 2 h and then incubated with HY-CHAL (3 µM) as in (**a**). * Indicates *p* < 0.05, significantly different from untreated control. ^#^ Indicates *p* < 0.05, significantly different from HY-CHAL.

Increased reactive oxygen species production is known to induce cell death and many anticancer agents are known to increase ROS levels and causing cell death [20,52]. To determine whether HY-CHAL induces ROS, cells were analyzed using the fluorochrome H_2_DCF-DA. The results revealed that HY-CHAL (3 µM) induced a fast (1 h) elevation of ROS (Figure 7a). To further confirm the relevance of these chemical species in the mechanism of cell death triggered by the chalcone, different antioxidants were used including ascorbic acid (100 µM), an analogue of vitamin E (trolox, 2 mM), *N*-acetyl-L-cysteine (NAC, 5 mM), catalase (500 units/mL) and glutathione (GSH, 5 mM). The results showed that catalase, NAC, and GSH were able to block apoptosis, being the tripeptide glutathione the most effective (Figure 7b). The levels of ROS increased ~2.5-fold after treatment with 3 μM HY-CHAL and these decreased in the presence of glutathione (Figure 7c). These results indicate that cell death induced by the chalcone is dependent on ROS generation.

Many reports have emphasized that chalcones may be an alternative and safe approach in the prevention and treatment of cancer. Previous studies have shown that isoliquiritigenin (4,2′,4′-trihydroxychalcone) decreases the viability of LNCaP and C4-2 prostate cancer cells, without sparing normal IEC-6 epithelial cells [53]. An analog containing a *N*-methylindole similar to HY-CHAL, JAI-51, selectively inhibits cancer cells in comparison to normal human nucleated blood cells and normal human skin fibroblasts cells [54]. This chalcone displayed antitumor activity in an in vivo glioblastoma model and has potential against taxane-resistant cancers [55]. Hydroxysafflor yellow A was well tolerated and non-toxic in humans and had reasonable pharmacokinetic properties [56]. A naturally occurring chalcone, millepachine, had antitumor activity in a HepG2 tumor-bearing xenograft mice model in vivo and showed less toxicity than doxorubicin [57]. Licochalcone A has been reported to inhibit cisplatin-induced kidney and liver damage [58] and similar results were obtained with isoliquiritigenin which was able to reduce chemotherapy-induced kidney and liver toxicity [59]. Interestingly, this chalcone induced a significant reduction in metastasic nodules in the lung in a renal mouse cell carcinoma model without causing leukocytopenia, a common feature of chemotherapeutic drugs [60]. Future studies to determine the potential of HY-CHAL in human medicine are needed to address its pharmacokinetic and pharmacodynamic properties.

## 3. Materials and Methods

### 3.1. Chemicals and Antibodies

The hybrid chalcone HY-CHAL was prepared by the condensation of α-bromoacrylic acid and a 5-aminoindole chalcone derivative as previously described [24]. HY-CHAL was dissolved in DMSO (dimethyl sulfoxide, Sigma, St. Louis, MO, USA) and kept under dark conditions at −20 °C. Before each experiment HY-CHAL was dissolved in culture media at 37 °C and the final concentration of DMSO did not exceed 0.3% (*v*/*v*). The inhibitor benzyloxycarbonyl-Val-Ala-Asp(OMe) fluoromethyl ketone (z-VAD-fmk, Calbiochem, Darmstadt, Germany) was from Calbiochem (Darmstadt, Germany). The inhibitors benzyloxycarbonyl-Asp(OMe)-Glu(O-Me)-Val-Asp(O-Me) fluoromethyl ketone (z-DEVD-fmk), benzyloxycarbonyl-Ile-Glu-Thr-Asp(OMe) fluoromethyl ketone (z-IETD-fmk), and benzyloxycarbonyl-Leu-Glu-His-Asp(OMe) fluoromethyl ketone (z-LEHD-fmk) were purchased from BD Pharmingen (San Diego, CA, USA). The inhibitors PD98059, U0126, SP600125, and SB203580 were purchased from Tocris (Bristol, UK). Acrylamide, bisacrylamide, ammonium persulfate and *N*,*N*,*N*′,*N*′-tetramethylethylenediamine were from Bio-Rad (Hercules, CA, USA). PVDF membranes and Immobilon Western Chemiluminiscent HRP Substrate were from Millipore (Billerica, MA, USA). All other chemicals were obtained from Sigma (St. Louis, MO, USA).

The primary antibodies used for Western blots were purchased from the following companies: anti-PARP [poly(ADP-ribose) polymerase,#551024, 1:5000 dilution], BD Pharmingen (San Diego, CA, USA), anti-caspase-3 (#ADI-AAP-113, 1:2000 dilution) from Enzo (Ann Arbor, MI, USA); anti-caspase-7 (#9494, 1:1000 dilution), anti-caspase-8 (#9746, 1:1000 dilution) and anti-caspase-9 (#9502S, 1:1000 dilution) from Cell Signaling Technology (Beverly, MA, USA); anti-cytochrome *c* (556433, 1:1000 dilution) from BD Pharmingen (San Diego, CA, USA); anti-Bcl-2 (#4223, 1:1000 dilution), anti-Bax (#2772, 1:1000 dilution), anti-Bcl-x_L_ (#2764, 1:1000 dilution), anti-Bak (#12105, 1:1000 dilution), anti-Mcl-1 (#4572, 1:1000 dilution), anti-Bid (#2002, 1:1000 dilution), anti-Bim (#2933, 1:1000 dilution), anti-JNK/SAPK (#9252, 1:1000 dilution), antiphospho-JNK/SAPK (phosphor T183 + Y185) (#9251, 1:1000 dilution), anti- p44/42 MAP Kinase (ERK1/2) (#9102, 1:1000 dilution), anti-Phospho-p44/42 MAPK (Erk1/2) (Thr202/Tyr204) (#9101, 1:1000 dilution), anti-p38MAPK (#9212, 1:1000 dilution), anti-phospho-p38MAPK (T180/Y182) (#9211, 1:1000 dilution); GSK-3β (#9315, 1:1000 dilution), anti-Phospho-GSK-3β (Ser9 (#9322, 1:1000 dilution), anti-Phospho-Akt (Ser473) (#9271, 1:1000 dilution), anti-β-actin (#4967, 1:1000 dilution); anti-p-p65 Phospho-NF-κB p65 (ser536) (#3033, 1:1000 dilution); anti-NF-κB p65 (#8242, 1:1000 dilution); anti-p21 (1:1000 dilution) antibodies from Cell Signaling Technology (Beverly, MA, USA). Anti-AKT (SC-1618, 1:1000 dilution) anti-c-myc (SC-40, 1:500 dilution) were from Santa Cruz Biotechnology (Santa Cruz, CA, USA). Anti-TRAIL (ab9959, 1:1000 dilution), anti-DR4 (ab8414, 1:1000 dilution) and anti-DR5 (ab47179, 1:1000 dilution) were from Abcam (Cambridge, UK). Anti-β-actin (clone AC-74, A2228, 1:1000 dilution) was from Sigma-Aldrich (Saint Louis, MO, USA); Horseradish peroxidase-conjugated secondary antibodies (NA9310 and NA9340, 1:10,000 dilution) were from GE Healthcare (Little Chalfont, UK).

### 3.2. Cell Culture and Viability Assays

Human melanoma cell lines SK-MEL-1 (ACC-303) and MEL-HO (ACC-62) were obtained from the German Collection of Microorganisms and Cell Cultures (Braunschweig, Germany). SK-MEL-1 are mostly round cells growing singly or in clumps in suspension [61]. They grow very slowly with a doubling time of several days, but with rapid medium acidification. Cells were described to produce pigment [62]. MEL-HO are epithelial-like adherent cells with doubling times of ca. 24 h. This cell line has significant tyrosinase mRNA expression and has been recently used as a model of TRAIL-sensitive cell lines that may still develop inducible TRAIL resistance [63]. Both cell lines have been reported to contain the BRAF V600E mutation [25,64]. Cells were cultured in RPMI 1640, 2 mM L-glutamine supplemented with 10% (*v*/*v*) heat-inactivated fetal bovine serum and 100 units/mL penicillin and 100 µg/mL streptomycin and maintained at 37 °C and 5% CO_2_ in a humidified atmosphere. Cell viability was assessed using 3-(4,5-dimethylthiazol-2-yl)-2,5-diphenyltetrazolium bromide (MTT) assay. Briefly, cells were seeded at a density of 5000 cells per well into a 96-well plate and incubated with increasing concentrations of HY-CHAL for 72 h. The supernatant was removed and 0.5 mg/mL MTT was added and incubated for 4 h at 37 °C. The reaction products were solubilized with sodium dodecyl sulfate (10% *w*/*v*) in 0.05 M HCl overnight under dark conditions. Absorbance was measured at 570 nm with a reference wavelength of 570 nm using an ELISA reader (Bio-Rad, Hercules, CA, USA). Each experimental condition was analyzed in triplicate. The IC_50_ values were determined graphically for each experiment by a nonlinear regression using the curve-fitting routine implemented within the software Prism 5.0 (GraphPad, La Jolla, CA, USA).

### 3.3. Evaluation and Quantification of Apoptosis

Fluorescence microscopy and flow cytometric analysis of propidium iodide-stained nuclei and of annexin V-FITC and propidium iodide-stained cells was carried out using a BD FACSVerse^TM^ cytometer (BD Biosciences, San Jose, CA, USA) were performed as previously described [65].

### 3.4. Western Blot Analysis

Immunoblot analysis of whole cell lysates and for cytosolic fraction was performed as previously described. For whole cell lysates, cell pellets were resuspended in lysis buffer [20 mM Tris-HCl (pH 7.4), 2 mM EDTA, 137 mM NaCl, 10% glycerol, 1% Triton X-100, 2 mM tetrasodium pyrophosphate, 20 mM sodium β-glycerophosphate, 10 mM sodium fluoride, 2 mM sodium orthovanadate], containing the protease inhibitors phenylmethylsulfonyl fluoride (PMSF, 1 mM), leupeptin, aprotinin, and pepstatin A (5 μg/mL each) for 15 min at 4 °C. Lysates were homogenized by a sonifier (five cycles) and centrifuged at 11,000× *g* for 10 min at 4 °C. Equal quantities of proteins from supernatants were boiled in SDS-sample buffer for 5 min before loading on an SDS-polyacrylamide gel (10% for MAPKs and 12.5% for caspases). Proteins were electrotransferred to poly(vinylidene difluoride) (PVDF) membranes and detected by enhanced chemiluminiscence. For subcellular fractionation, cells were washed twice with PBS and then resuspended in ice-cold buffer [20 mM HEPES (pH 7.5), 250 mM sucrose, 10 mM KCl, 1.5 mM MgCl_2_, 1 mM EDTA, 1 mM EGTA, 1 mM dithiothreitol, 0.1 mM PMSF, and 5 μg/mL aprotinin, leupeptin, and pepstatin A]. After 15 min on ice, cells were lysed by pushing them several times through a 22-gauge needle, and the lysate was centrifuged at 1000× *g* for 5 min at 4 °C to eliminate nuclei and unbroken cells. This pellet was used as a nuclear fraction. The supernatant fraction was centrifuged at 105,000× *g* for 45 min at 4 °C, and the resulting supernatant was used as the soluble cytosolic fraction.

### 3.5. Assay of Caspase Activity

Caspase activity was determined in cell lysates using specific colorimetric substrates. Briefly, cells were treated with increasing concentrations of HY-CHAL for 12 or 24 h, harvested by centrifugation at 1000× *g* for 5 min at 4 °C and washed with PBS, and the cell pellets were kept on ice. The cells were resuspended in lysis buffer (50 mM HEPES, pH 7.4, 0.1 mM EDTA, 1 mM dithiothreitol, 0.1% Chaps) and held on ice for 5 min. Lysates were centrifuged at 17,000× *g* for 10 min at 4 °C and the supernatants were collected. The total protein concentration in the supernatants was determined using the Bradford dye-binding assay. Equal quantities of protein from different treatments were used, and the assays were set up on the ice. The net increase of absorbance at 405 nm after incubation at 37 °C was indicative of enzyme activity. Specific colorimetric substrates for caspase-3/7, −8 and −9 activities were DEVD-*p*NA (*N*-acetyl-Asp-Glu-Val-Asp-*p*-nitroaniline), IETD-*p*NA (*N*-acetyl-Ile-Glu-Thr-Asp-*p*-nitroaniline) and LEHD-*p*NA (*N*-acetyl-Leu-Glu-His-Asp-*p*-nitroaniline), respectively.

### 3.6. Analysis of Mitochondrial Membrane Potential ΔΨ_m_ and Detection of Intracellular Reactive Oxygen Species (ROS)

The membrane potential and intracellular ROS production were determined by flow cytometry. The fluorochrome 5,5′,6,6′-tetrachloro-1,1′,3,3′-tetraethylbenzimidazolylcarbocyanine iodide (JC-1, 5 μg/mL) was used to measure mitochondrial membrane depolarization. The fluorochrome 2′,7′-dichlorodihydrofluorescein diacetate (H_2_-DCF-DA, 10 μM) was used to determine ROS.

### 3.7. Statistical Analysis

Two-way ANOVA was used to test for differences between concentrations and between cell types, with a Tukey post-hoc test used to compare between treatment concentrations and the control. The standard significance level (s.l.) of 0.05 was used. The GraphPad Prism 5 software was used.

## 4. Conclusions

We have evaluated the effects of a specific chalcone containing indole and pyridine heterocyclic rings on cell viability and apoptotic pathways in human melanoma cell lines. This hybrid chalcone, HY-CHAL, was a potent cytotoxic compound and an apoptotic inducer in SK-MEL-1 and MEL-HO melanoma cells. The fact that both melanoma cell lines were sensitive to HY-CHAL might be interesting given that melanoma is the most aggressive and lethal form of skin cancer and frequently resists chemotherapy. Here we explored the mechanism of cell death by investigating apoptotic pathways. In both cell lines, the antiproliferative effect of HY-CHAL was associated with the release of cytochrome *c* and a mechanism dependent of caspases. Futures studies will be necessary to determine whether additional pathways of cell death are involved in SK-MEL-1 and MEL-HO melanoma cells. The sensitivity of melanoma cells to HY-CHAL suggests that it should be considered for further preclinical and in vivo testing. The exploration of the signal transduction pathway of cell death using SK-MEL-1 cells as a model revealed that this compound induced (i) cell death by a mechanism that involves the intrinsic and the extrinsic pathways, (ii) activation of the MAPK pathway and the blockade of MEK/ERK cascade amplified the cell death, and (iii) downregulation of β-catenin, c-myc, p21^Cip1/WAF1^, and Mcl-1 and (iv) inhibition of NF-κB. In addition, cell death was dependent on the generation of reactive oxygen species since HY-CHAL induced an increase in ROS levels, and pre-incubation with glutathione suppressed the increase of ROS and inhibited significantly the cell death. The studies described here may open new avenues of research to understand the safety and full potential of HY-CHAL in human health.

## Data Availability

Data is contained within the article or supplementary material.

## Supplementary Materials

The following are available online at https://www.mdpi.com/article/10.3390/ijms222413462/s1.
