# Supplementary material for: Apoptosis Pathways Triggered by a Potent Antiproliferative Hybrid Chalcone on Human Melanoma Cells
Source: Int J Mol Sci. 2021 Dec 15;22(24):13462. doi: 10.3390/ijms222413462 (PMC8706831; doi:10.3390/ijms222413462)
Supplement: Supplementary file 1 [file ijms-22-13462-s001.zip › ijms-1464915-supplementary.pdf]

Supplementary Materials

Figure S1. HY-CHAL induced caspase cascade in MEL-HO cells.

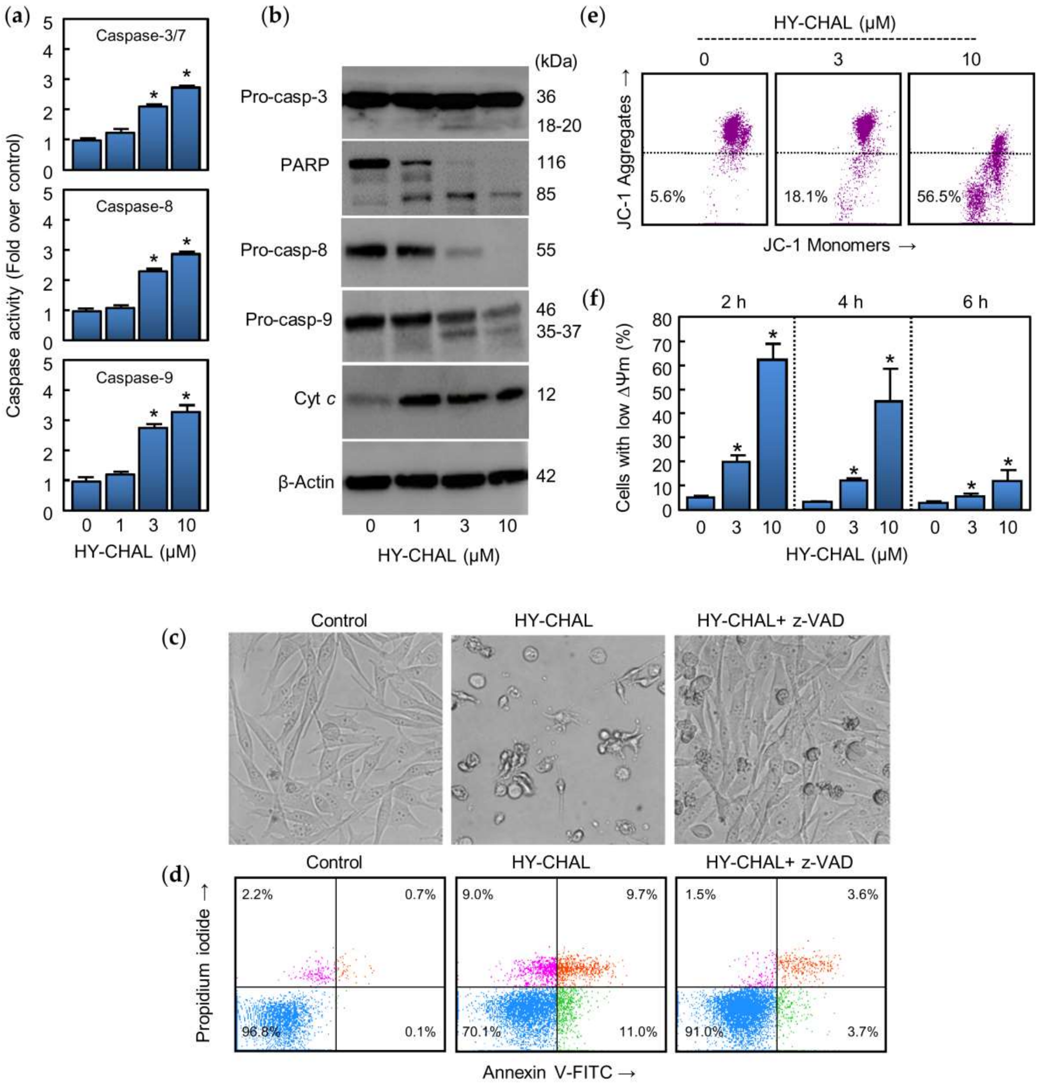

Figure S1. HY-CHAL induced caspase cascade in MEL-HO cells. (**a**) Cells were treated with the specified concentrations of HY-CHAL, harvested at 24 h and total cell lysates were assayed for caspase-3/7, -8, and -9 activities. Results are expressed as factorial increases in caspase activity compared with the control. Values represent means ± SE of two independent experiments each performed in triplicate; (**b**) Cells were treated as in (**a**) and whole cell lysates or cytosolic fractions (in the case of cytochrome *c*) were analyzed by immunoblotting. β-Actin was used as a loading control; (**c**) Cells were pretreated with 100 μM z-VAD-fmk for 1 h and then incubated in the absence or in the presence of 3 μM HY-CHAL for 24 h and images were obtained with an inverted phase-contrast microscope; (**d**) Cells were treated as in (**c**) and analyzed by flow cytometry after staining with annexin V-FITC and propidium iodide; (**e**) Cells were incubated with the indicated concentrations of HY-CHAL for 2 h and $\Delta\Psi_m$ analyzed by flow cytometry after staining with the JC-1 probe; (**f**) Cells were incubated in control conditions or in the presence of the indicated concentrations of HY-CHAL for the specified times, and the percentage of cells with reduced $\Delta\Psi_m$ was quantified by flow cytometry using JC-1. * Indicates $p < 0.05$ for comparison with untreated control.

Whole Western blots. Uncropped western blots

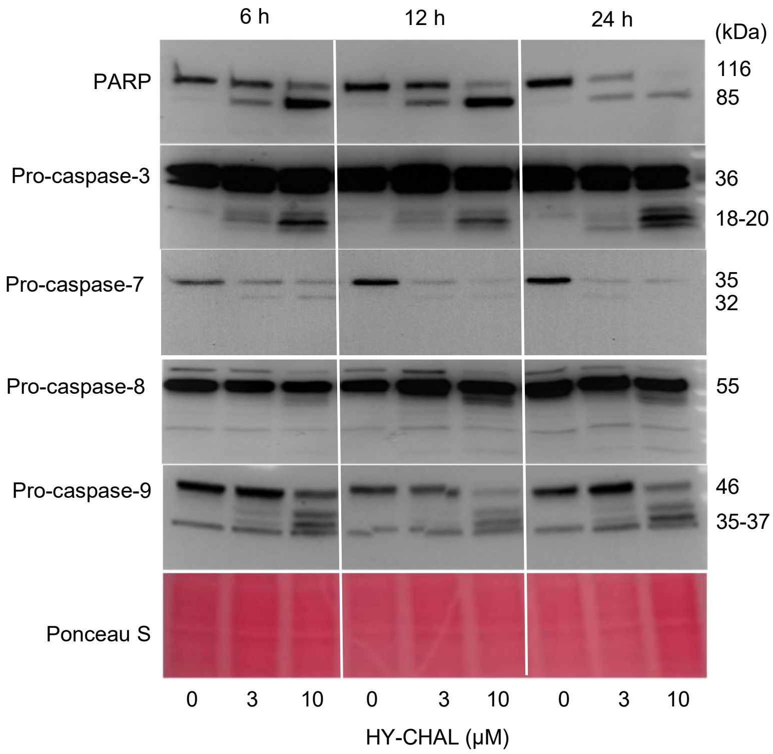

PARP

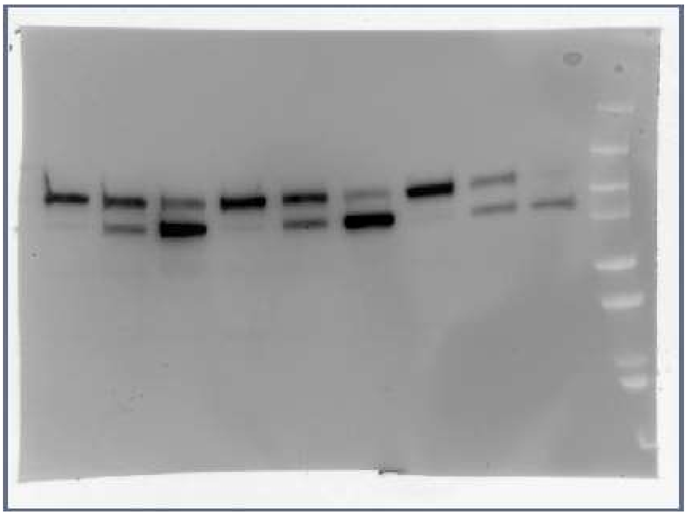

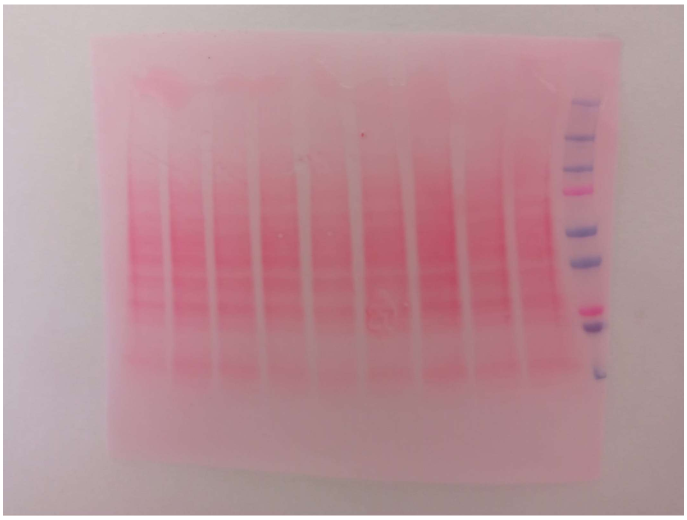

Pro-caspase-3

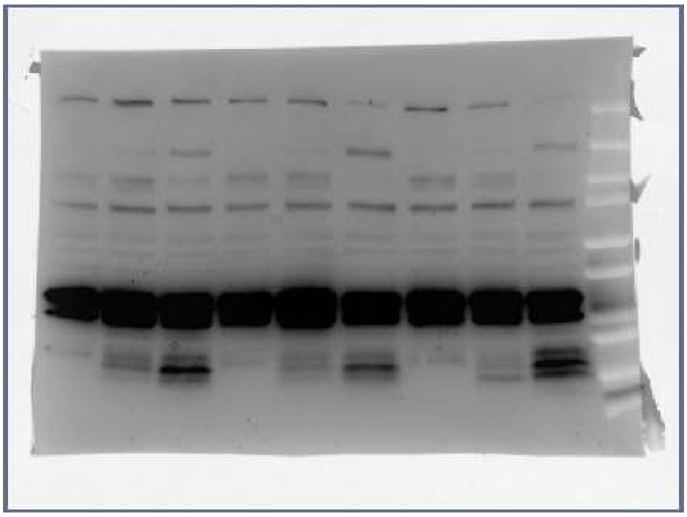

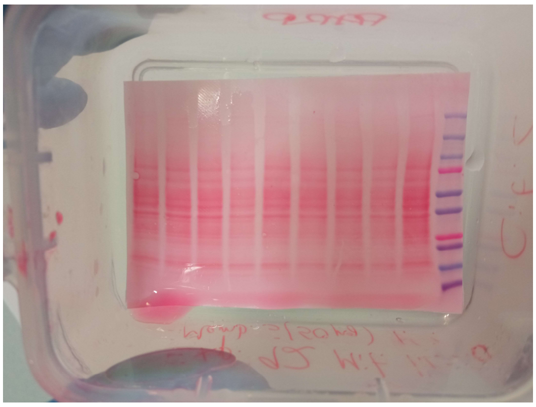

Pro-caspase-7

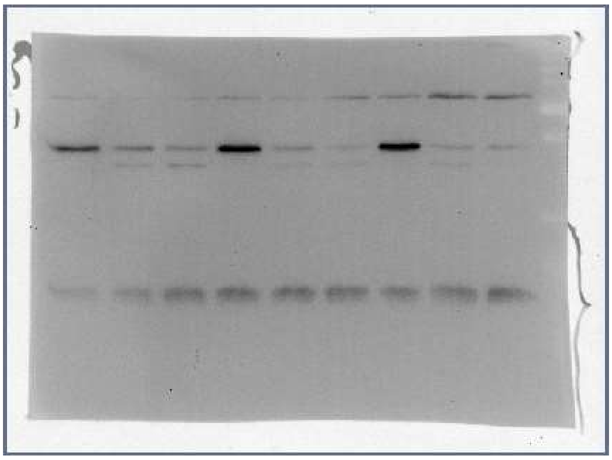

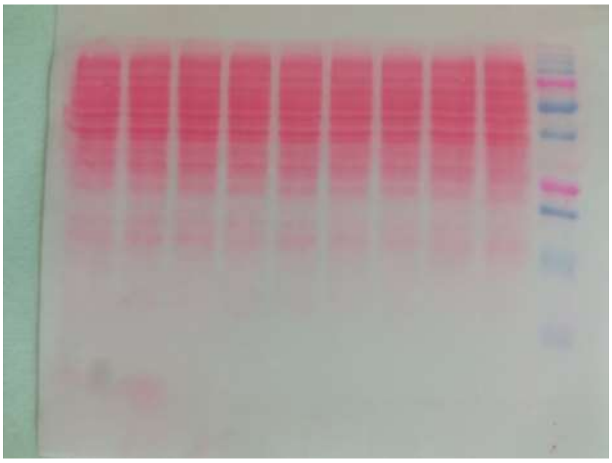

Pro-caspase-8

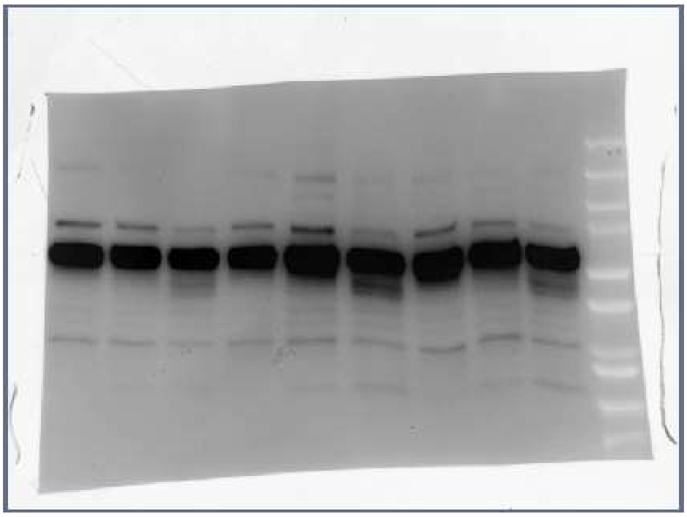

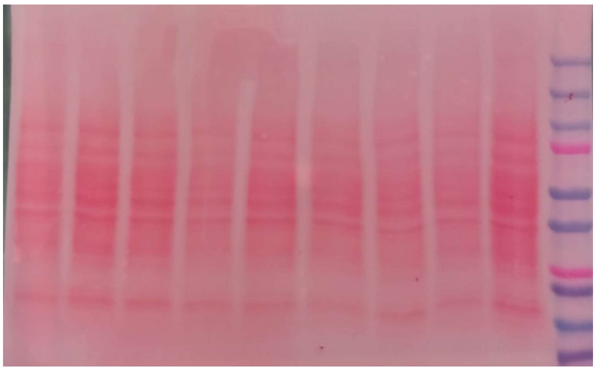

Pro-caspase-9

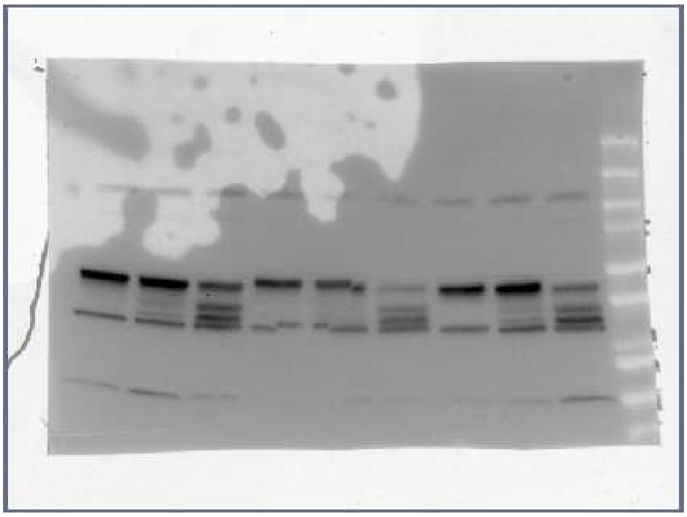

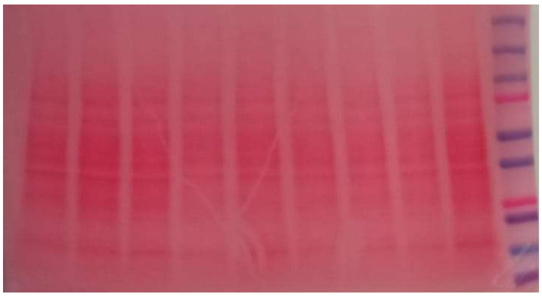

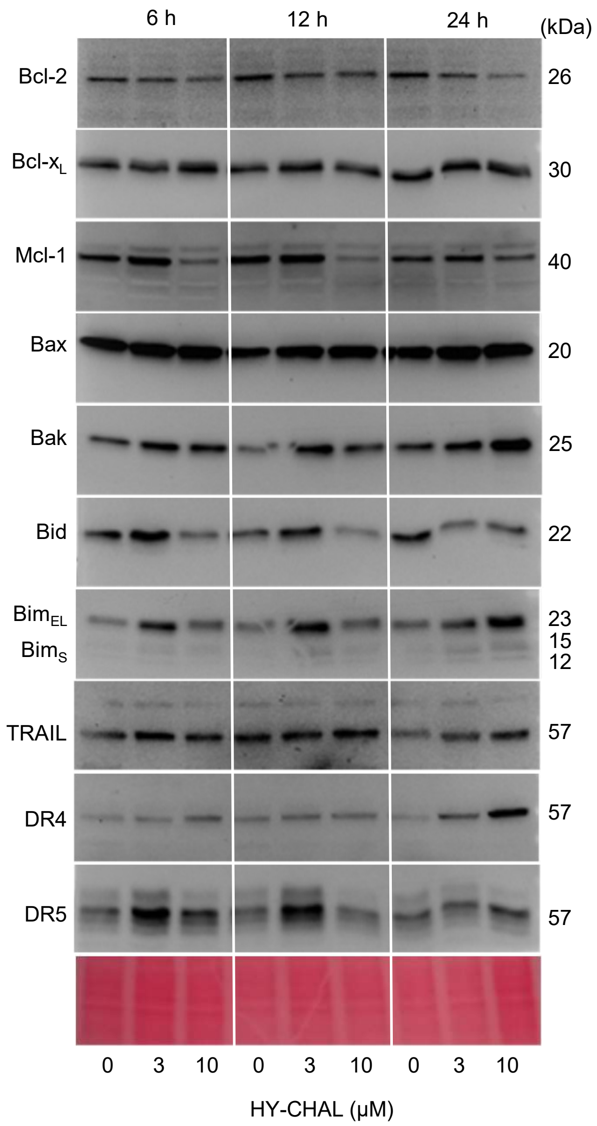
6 h
12 h
24 h
(kDa)
Bcl-2
26
Bcl-x$_L$
30
Mcl-1
40
Bax
20
Bak
25
Bid
22
Bim$_{EL}$
Bim$_S$
23
15
12
TRAIL
57
DR4
57
DR5
57
0
3
10
0
3
10
0
3
10
HY-CHAL (μM)

Bcl-2

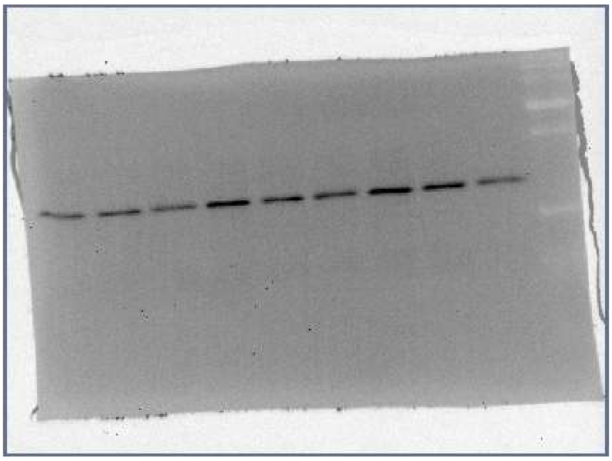

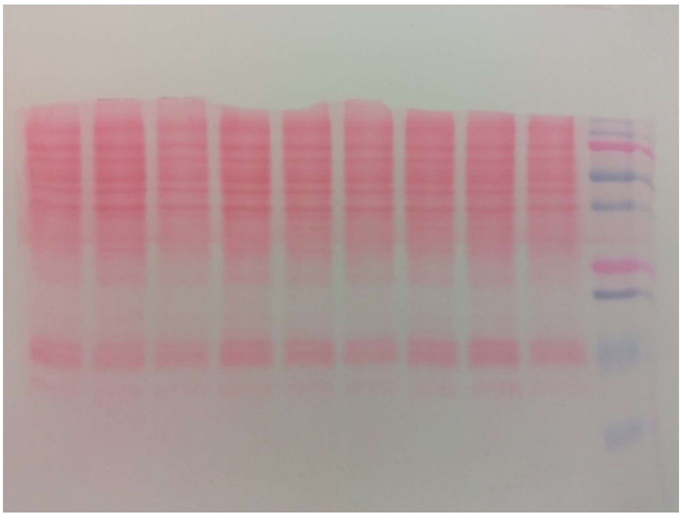

Bcl-x$_L$

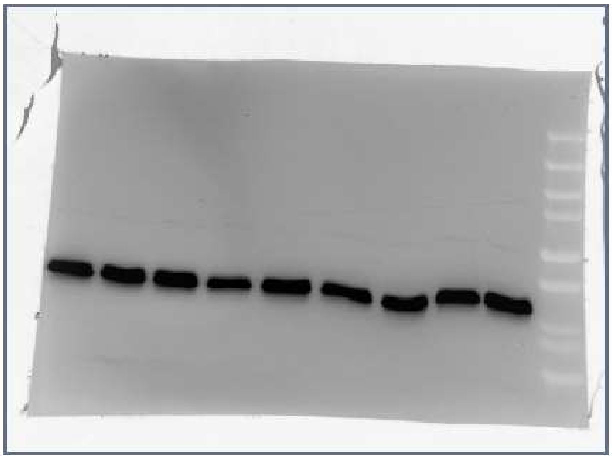

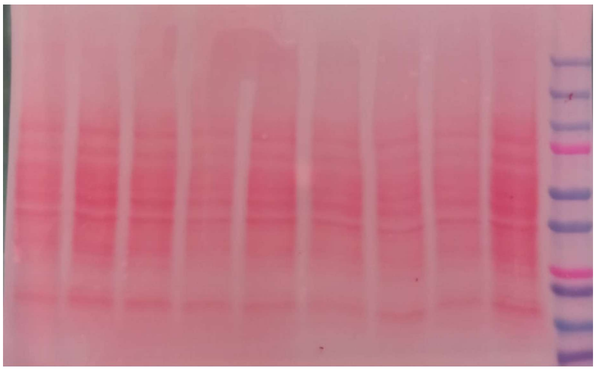

Mcl-1

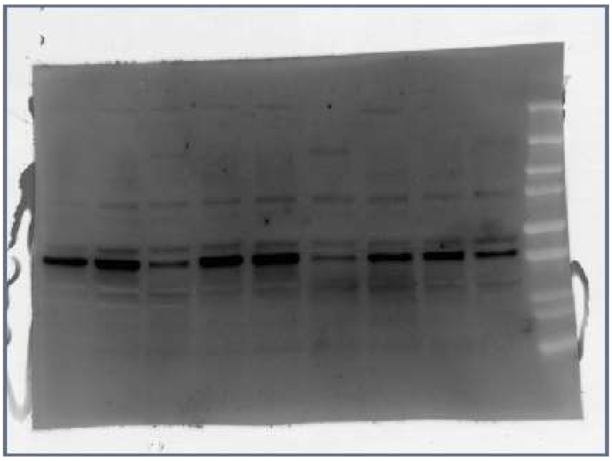

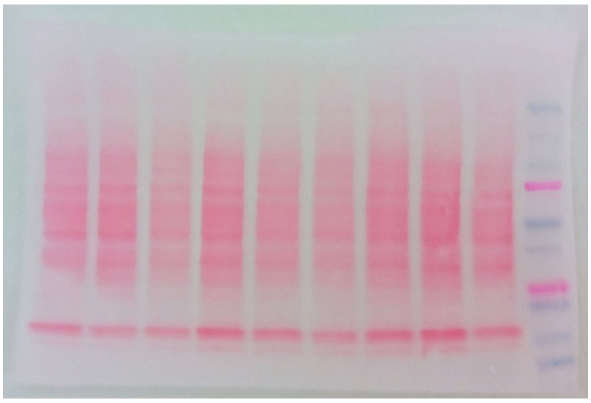

Bax

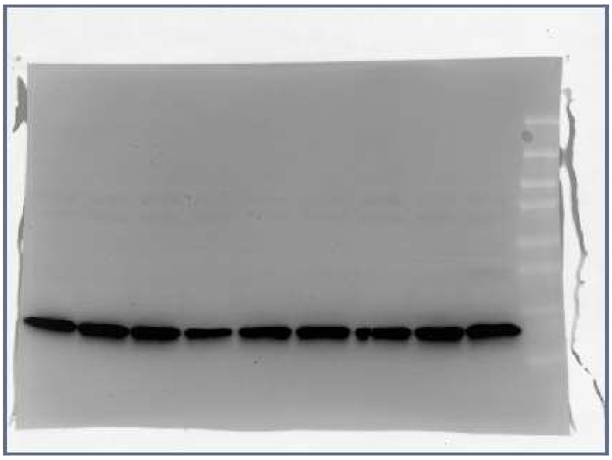

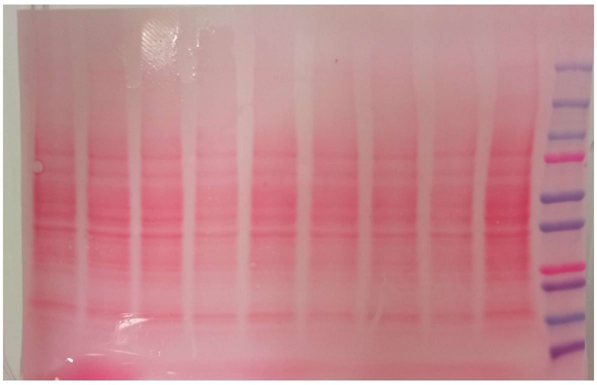

Bak

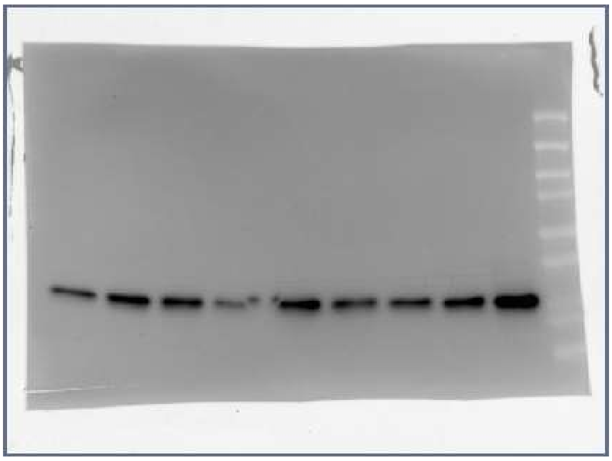

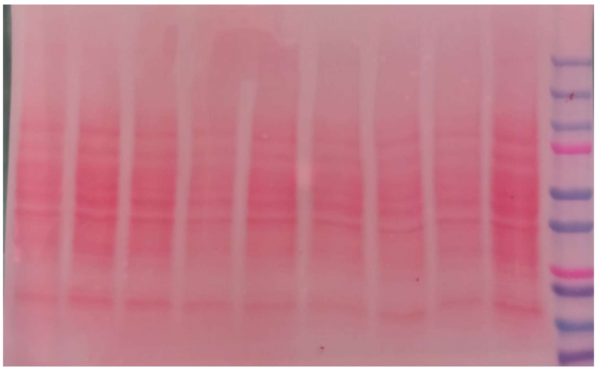

Bid

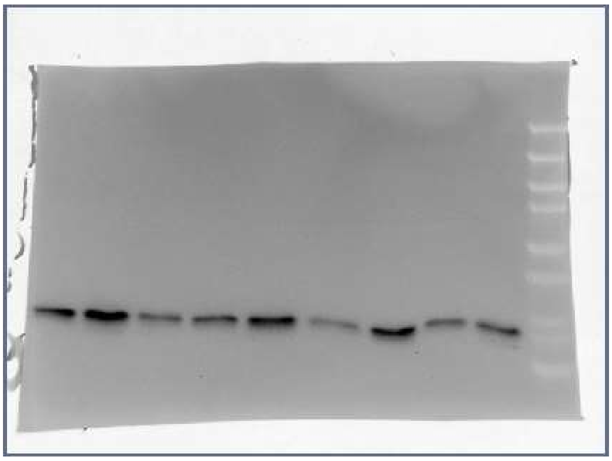

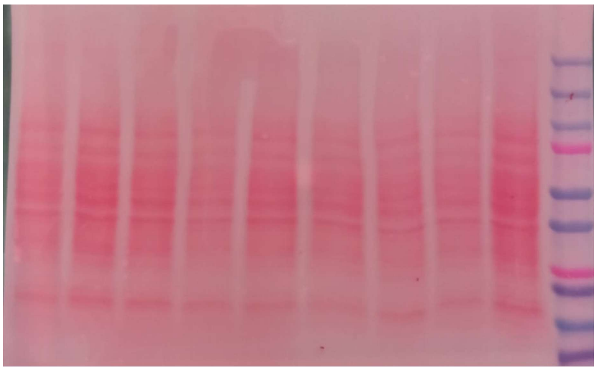

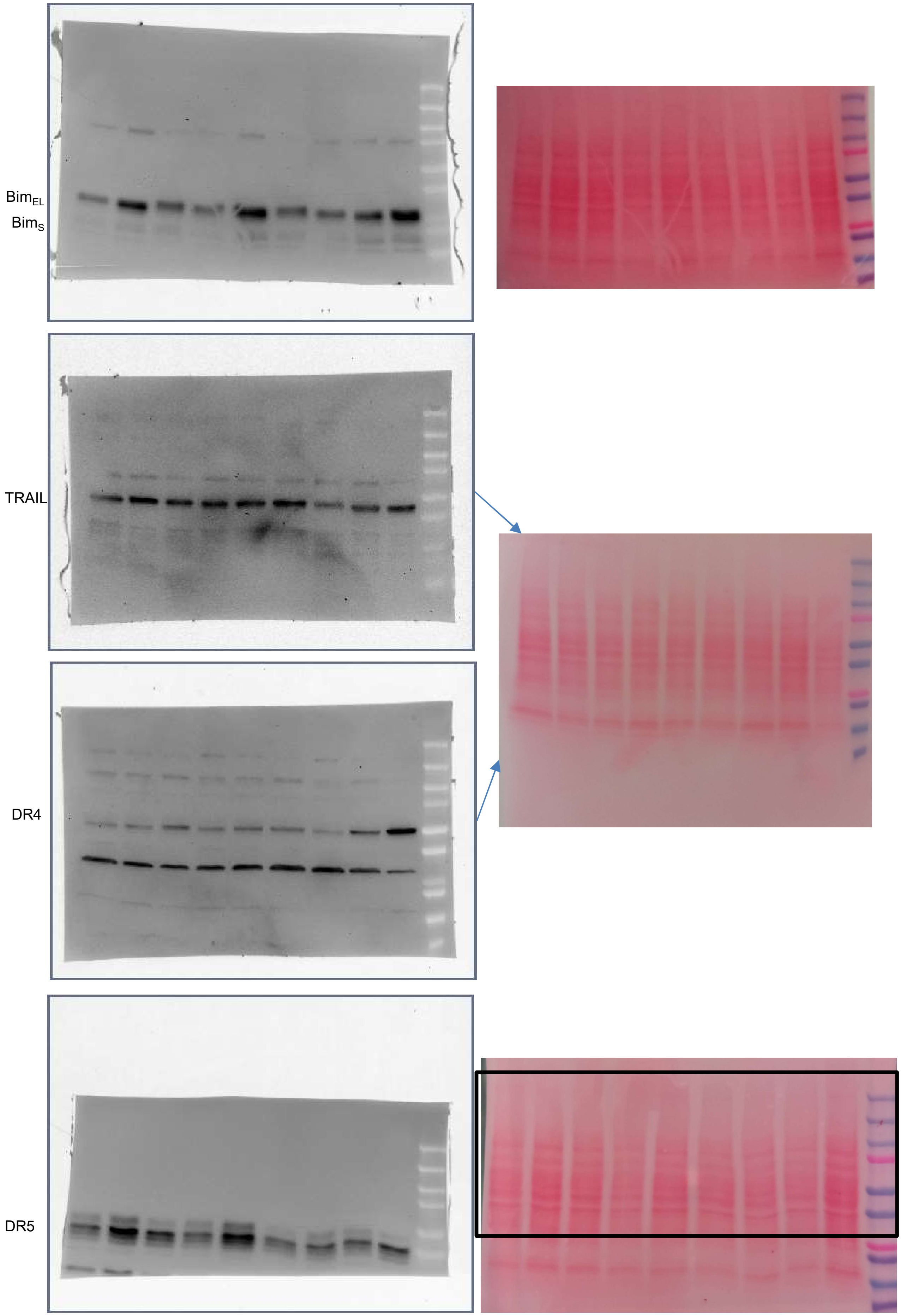
Bim_{EL}
Bim_S
TRAIL
DR4
DR5

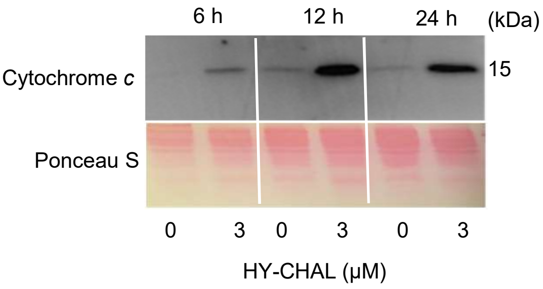
6 h
12 h
24 h
(kDa)
Cytochrome *c*
15
Ponceau S
0
3
0
3
0
3
HY-CHAL (μM)

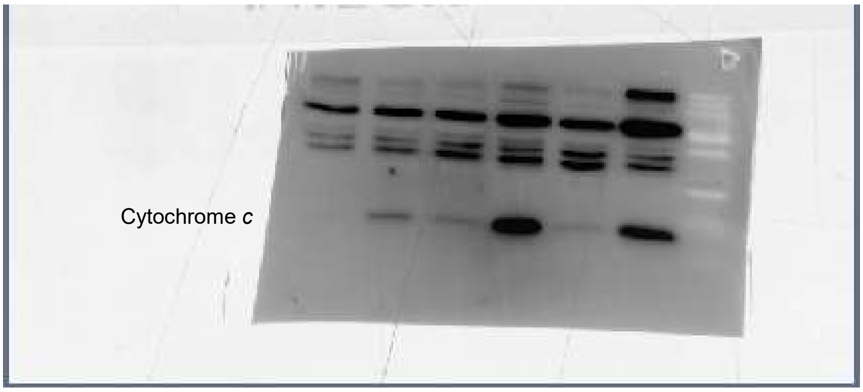
Cytochrome *c*

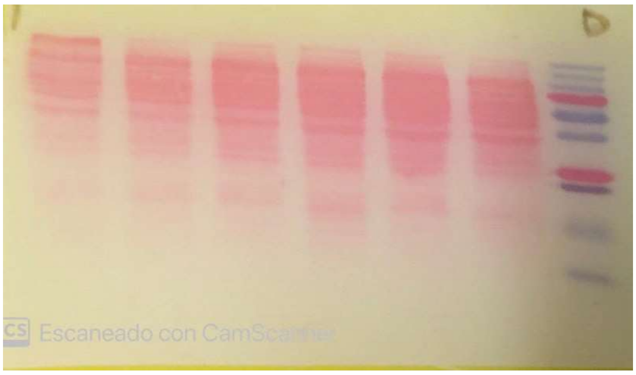
Escaneado con CamScanner

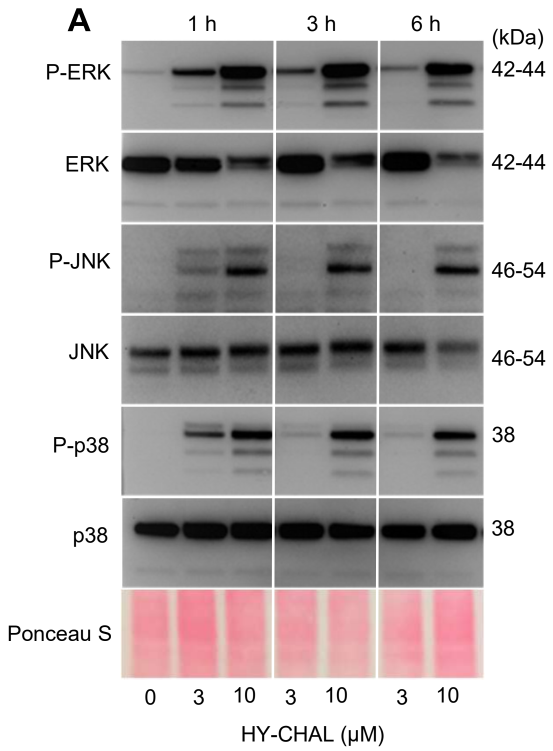

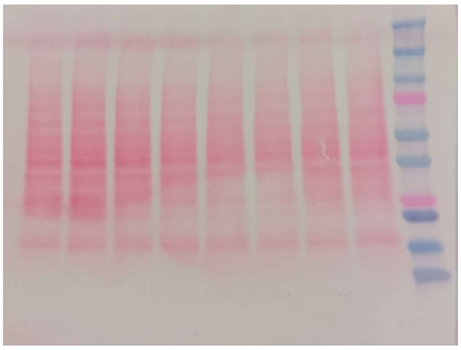

P-ERK

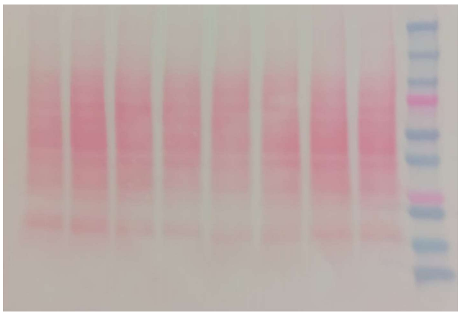

P-JNK

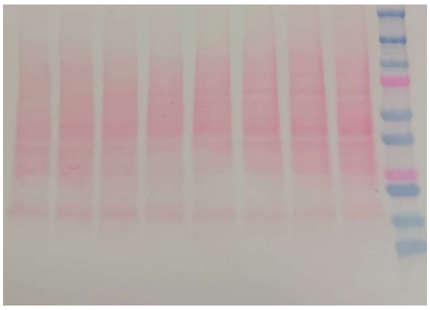

P-p38

P-ERK

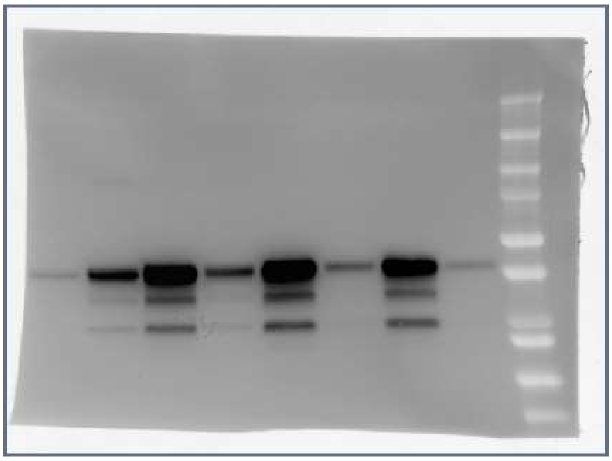

ERK

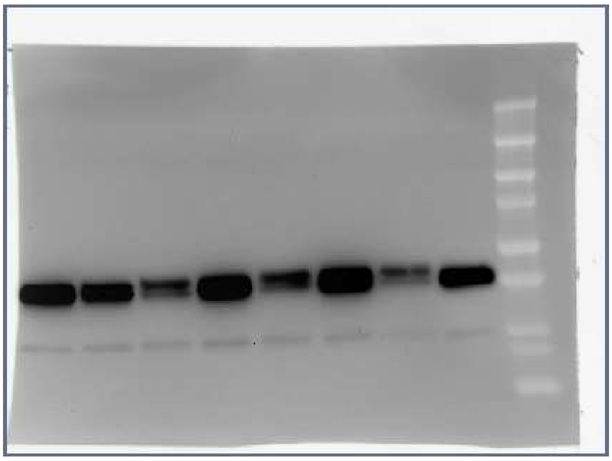

Last line, TPA, is not included in Figure 5a.

P-JNK

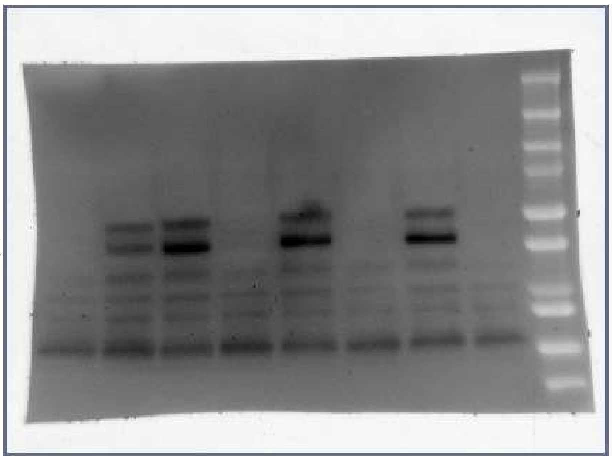

Last line, anisomycin, is not included in Figure 5a.

JNK

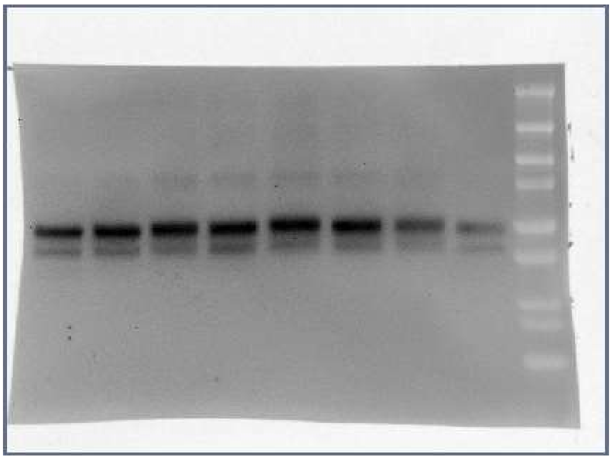

Last line, anisomycin, is not included in Figure 5a.

P-p38

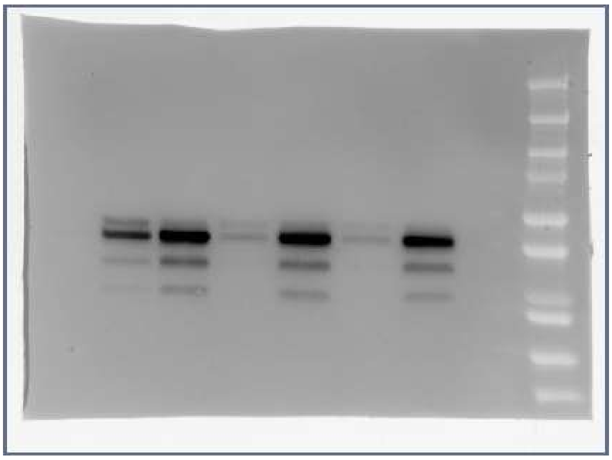

Last line, anisomycin, is not included in Figure 5a.

p38

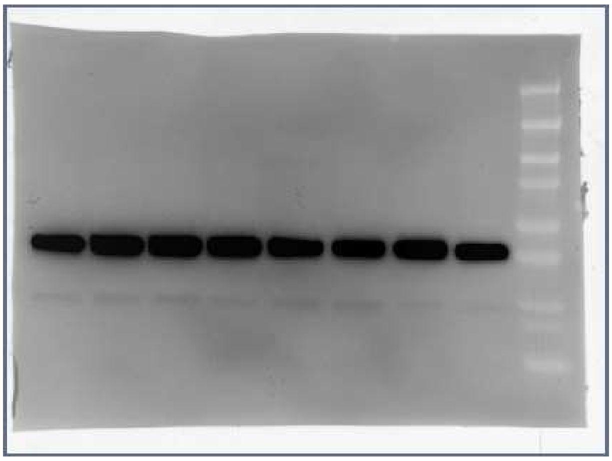

Last line, anisomycin, is not included in Figure 5a.

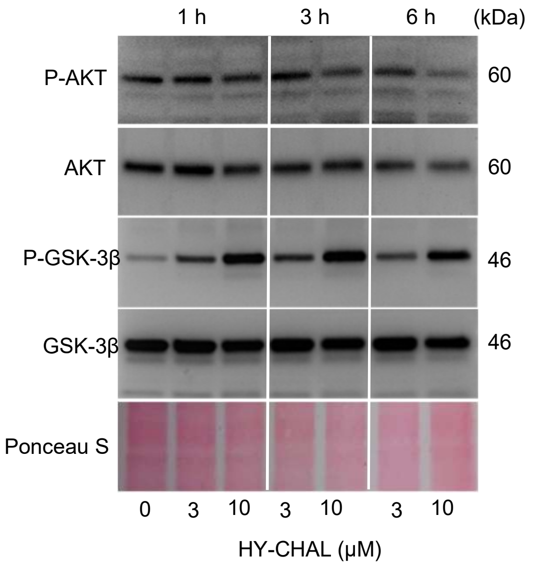

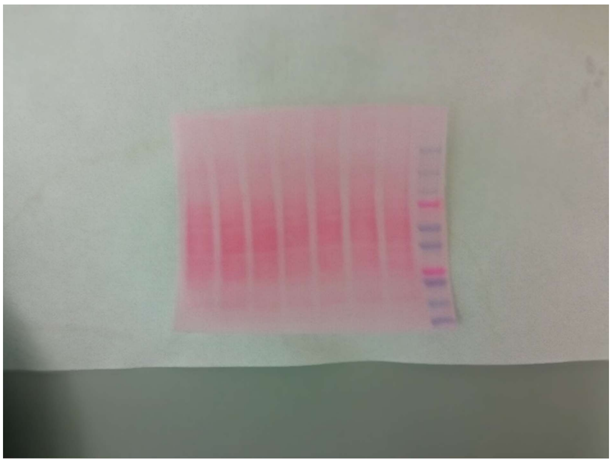

P-AKT

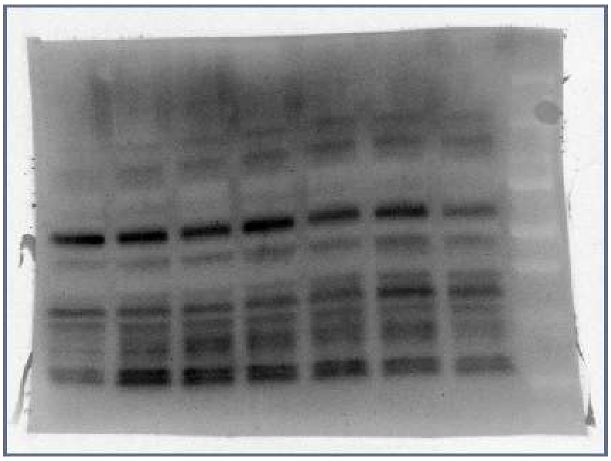

AKT

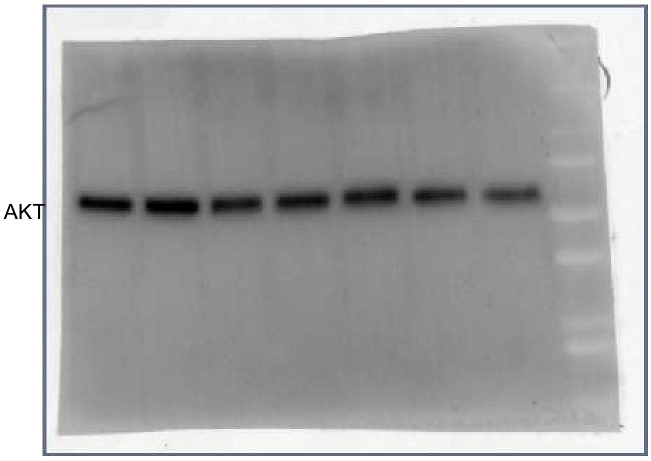

P-GSK-3β

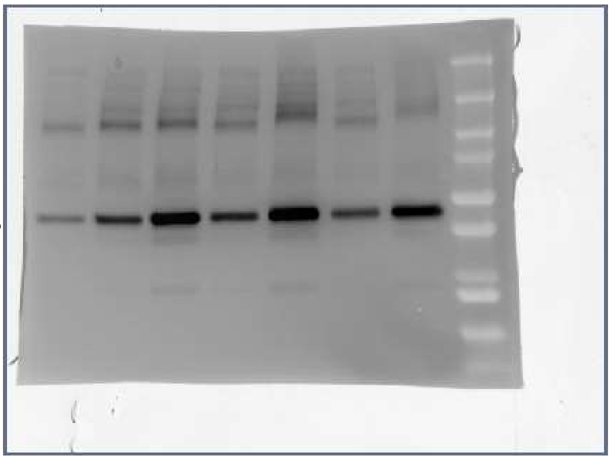

GSK-3β

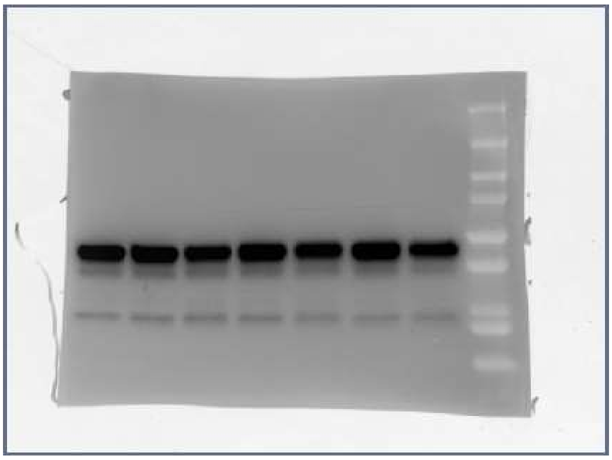

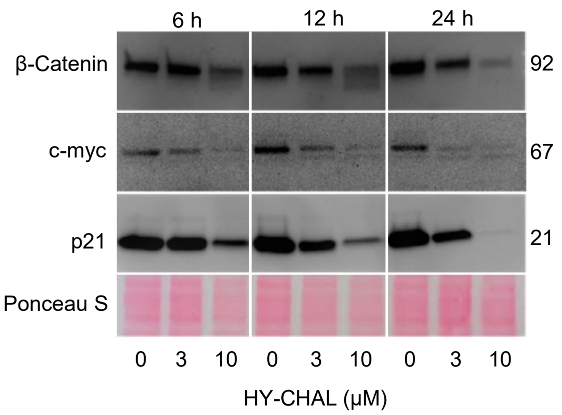

β-Catenin

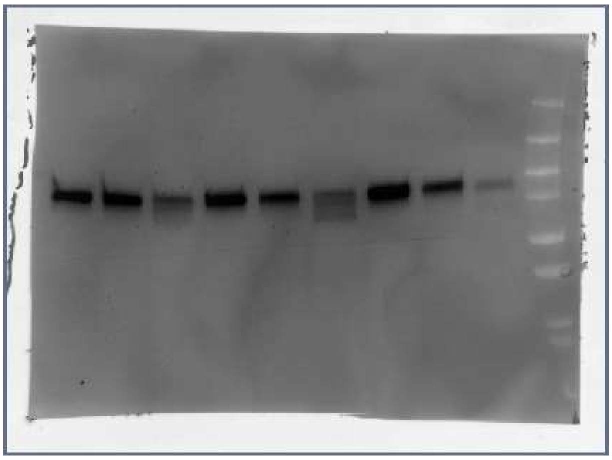

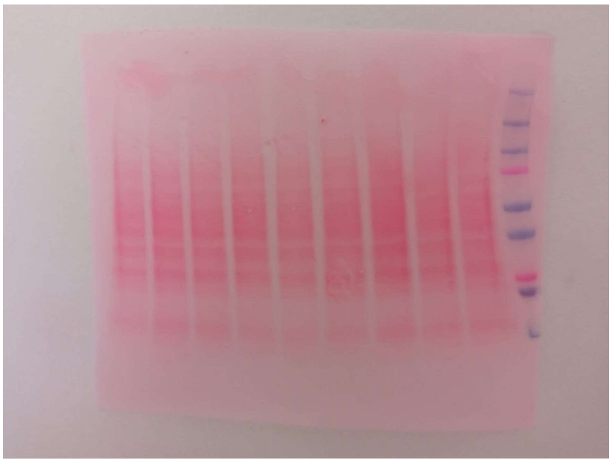

c-myc

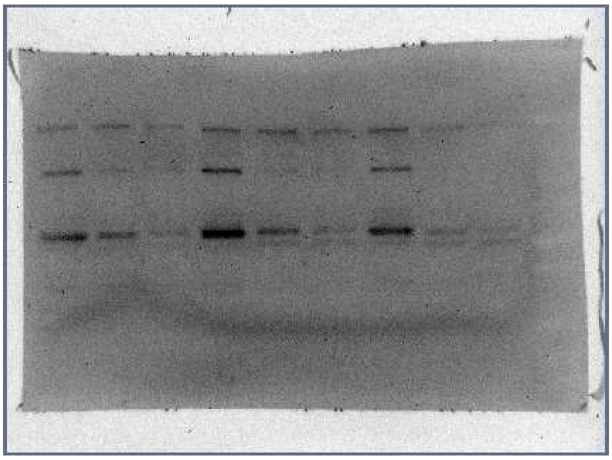

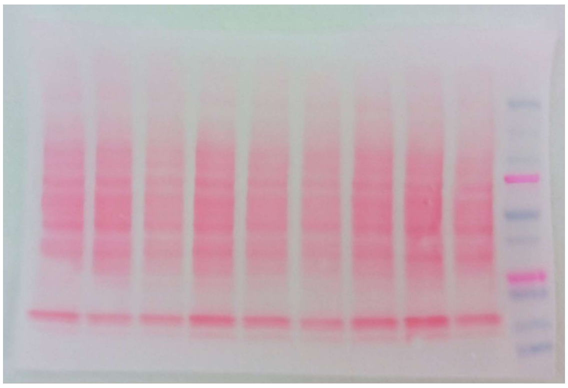

p21

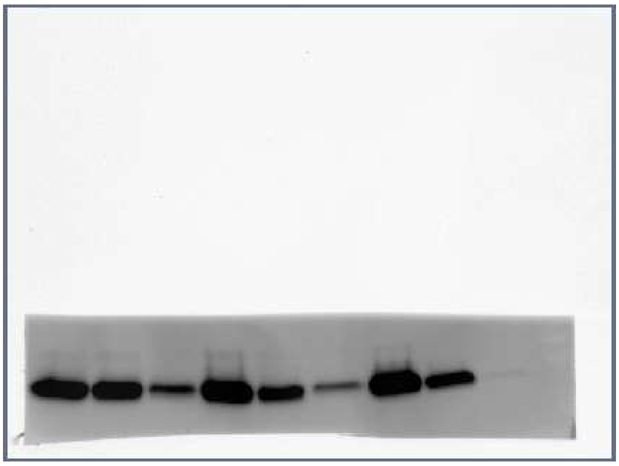

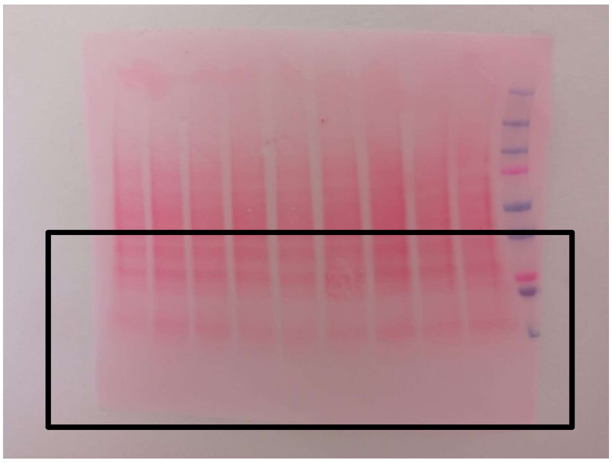

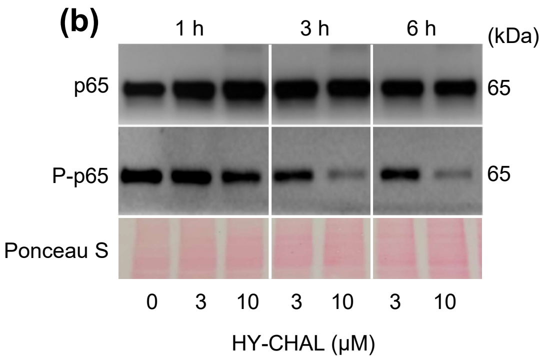

p65

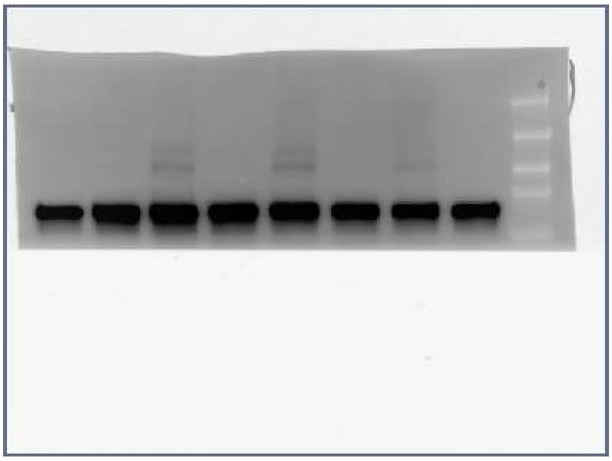

P-p65

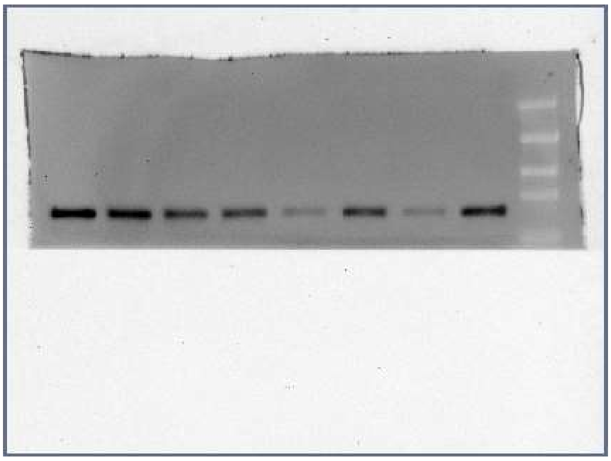

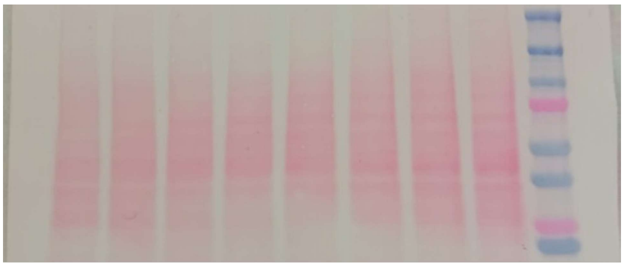

Last line (anisomycin) is not included.in Figure 6b.

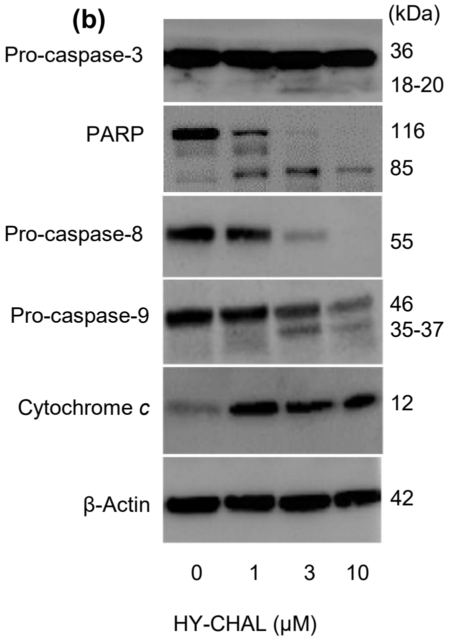

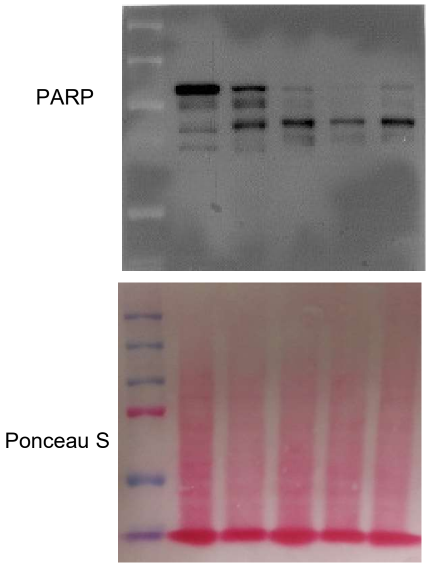

Last line is not included.in Figure S1b.
Ponceau S staining is not included.

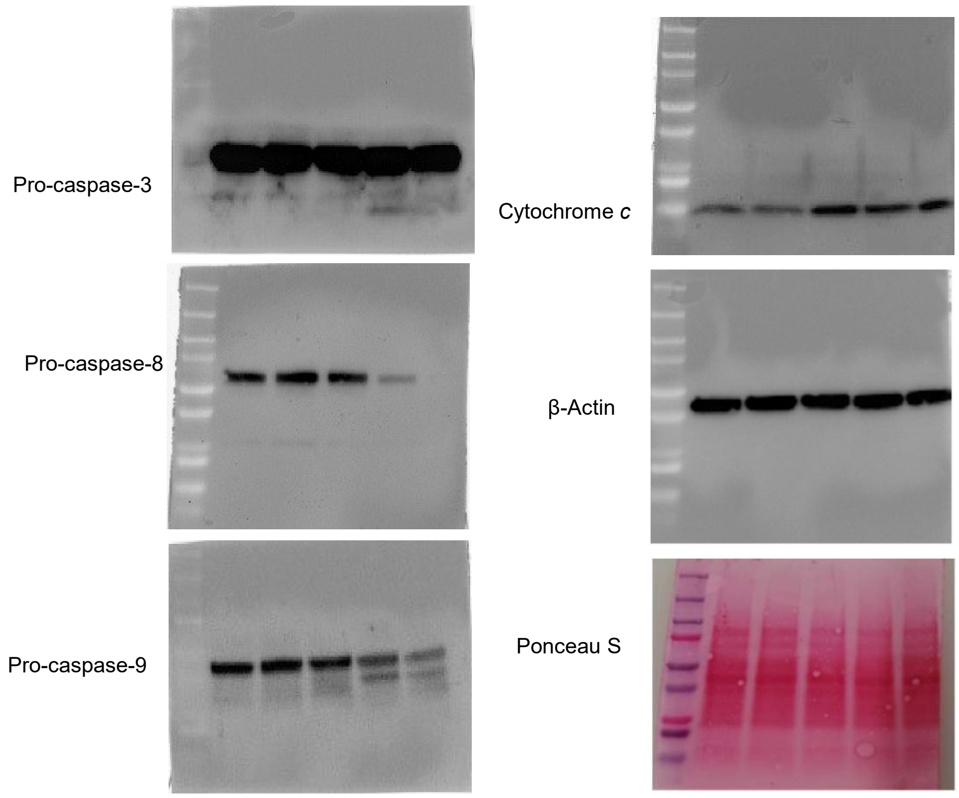

First line (control, equal to line 2) is not included in Figure S1b.
Ponceau S staining is not included.
